# Heat, Brain, and Mental Health: Biological Mechanisms Underlying Climate-Related Psychiatric Outcomes

**DOI:** 10.3390/biology15141165

**Published:** 2026-07-16

**Authors:** Julio Torales, Iván Barrios, Marcelo O’Higgins, Tomás Caycho-Rodríguez, Antonio Ventriglio, João Mauricio Castaldelli-Maia

**Affiliations:** 1 Grupo de Investigación sobre Epidemiología de los Trastornos Mentales, Psicopatología y Neurociencias, Facultad de Ciencias Médicas, Universidad Nacional de Asunción, San Lorenzo 111421, Paraguay; marcelo.g.ohiggins@gmail.com; 2 Vicerrectoría de Investigación y Postgrado, Universidad de Los Lagos, Osorno 5290000, Chile; 3 Facultad de Ciencias de la Salud, Universidad Sudamericana, Pedro Juan Caballero 130114, Paraguay; ivanjuan2013@gmail.com; 4 Cátedra de Bioestadística, Filial Santa Rosa del Aguaray, Facultad de Ciencias Médicas, Universidad Nacional de Asunción, Santa Rosa del Aguaray 021801, Paraguay; 5 Facultad de Psicología, Universidad Científica del Sur, Lima 15067, Peru; tcaycho@cientifica.edu.pe; 6 Department of Clinical and Experimental Medicine, University of Foggia, 71121 Foggia, Italy; a.ventriglio@libero.it; 7 Department of Psychiatry, University of São Paulo, São Paulo 05403903, SP, Brazil; jmcmaia2@gmail.com

**Keywords:** climate change, heat exposure, mental health, psychiatry, thermoregulation, neuroinflammation, oxidative stress, sleep, psychopharmacology, geopsychiatry

## Abstract

Heat exposure is an increasingly important climate-related determinant of mental health, yet the biological mechanisms linking heat to psychiatric outcomes remain insufficiently integrated. This narrative review synthesizes evidence from climate medicine, thermoregulation, neuroscience, psychoneuroimmunology, sleep research, psychopharmacology, epidemiology, and psychiatry to examine how acute and chronic heat exposure may influence mental health. Heat acts as a multi-system biological stressor by challenging thermoregulation, increasing autonomic and cardiovascular strain, disrupting hydration, impairing sleep and circadian rhythms, activating stress-response systems, and contributing to neuroinflammation, oxidative stress, mitochondrial dysfunction, blood–brain barrier disruption, and neurotransmitter alterations. These pathways may interact with psychotropic medication exposure and impaired behavioral thermoregulation, particularly among people with severe mental illness, older adults, individuals receiving lithium, people with cognitive impairment, substance use disorders, outdoor workers, and those living in poorly cooled environments. Heat-related psychiatric vulnerability is therefore transdiagnostic but heterogeneous, with relevance to distress, depression, anxiety, mood instability, psychosis, substance-related crises, cognitive dysfunction, delirium, and suicide-related outcomes. The review also distinguishes pathways supported by stronger human evidence from mechanisms supported mainly by experimental, preclinical, severe-spectrum, or indirect evidence. It proposes an integrative biological model and an evidence-informed translational agenda focused on exposure assessment, biomarkers, sleep and circadian monitoring, medication safety, risk prediction, physiological adaptation, heat-mitigation strategies, heat-health action plans, and implementation-oriented evaluation.

## Simple Summary

Climate change increases human exposure to heatwaves, high nighttime temperatures, indoor heat, and occupational heat. Although heat is often studied in relation to cardiovascular, renal, and respiratory health, its effects on mental health are clinically important. This review examines the effects of heat on the brain and body through biological pathways relevant to psychiatry. These include thermoregulatory strain, autonomic and cardiovascular activation, dehydration, neuroinflammation, oxidative stress, mitochondrial dysfunction, blood–brain barrier disruption, stress system activation, sleep and circadian disruption, neurotransmitter changes, and psychotropic medication-related vulnerability. These mechanisms may contribute to psychological distress, depression, anxiety, mood instability, psychosis, substance-related crises, cognitive dysfunction, delirium, and suicide-related outcomes. The review also emphasizes that vulnerability is shaped by age, comorbidity, psychiatric diagnosis, medication exposure, housing, occupational exposure, access to cooling, health care capacity, and social support. At the same time, it recognizes that some heat responses may involve adaptation and resilience when exposure is moderate and recovery is adequate. Understanding heat as a multi-system biological stressor can help psychiatry develop better prevention strategies, safer prescribing practices, heat-mitigation approaches, and climate-informed mental healthcare.

## 1. Introduction

Climate change is increasingly recognized as a major determinant of human health, with heat exposure being one of its most direct and biologically consequential pathways. The Sixth Assessment Report of the Intergovernmental Panel on Climate Change identifies climate-related hazards as growing threats to physical health, mental health, and well-being, particularly through extreme heat, displacement, food insecurity, and health system disruption [1]. Similarly, recent global climate-health assessments have emphasized that heat-related morbidity and mortality are increasing as the frequency, intensity, and duration of extreme heat events increase across regions [2]. Mental health has historically received less attention than cardiovascular, renal, respiratory, and infectious disease outcomes in climate-health research; however, the World Health Organization has explicitly called for the integration of mental health into climate adaptation and public health planning [3]. Conceptual models proposed more than a decade ago have already framed climate change as a multi-pathway mental health risk, operating through direct exposure to extreme weather, indirect ecological and socioeconomic disruption, and broader existential and community-level stressors [4]. Within this broader framework, heat exposure deserves focused biological attention because it is not merely an external environmental condition but a physiological stressor capable of altering the brain, endocrine, immune, sleep, and behavioral regulation.

Growing epidemiological literature supports an association between elevated ambient temperatures and adverse mental health outcomes. Systematic reviews have reported links between high temperatures or heatwaves and a range of psychiatric endpoints, including psychological distress, mental health-related emergency department visits, psychiatric admissions, substance-related outcomes, exacerbation of severe mental disorders, and suicide mortality [5,6,7]. Earlier reviews found the most consistent evidence for increased suicide risk during periods of elevated temperature, whereas evidence for other diagnostic categories was more heterogeneous because of differences in exposure definitions, lag structures, outcome measures, and study settings [6]. More recent synthesis has strengthened the evidence base by showing that ambient temperature is associated with mental health-related morbidity and mortality in diverse populations [7]. Large population-based studies have also contributed to this field. Obradovich et al. showed that short-term exposure to more extreme weather, multiyear warming, and tropical cyclone exposure were associated with worsened mental health in the United States [8]. Burke et al. reported that a 1 °C increase in monthly average temperature was associated with higher suicide rates in both the United States and Mexico, and also found evidence of worsened depressive language on social media during warmer periods [9]. A recent systematic review and meta-analysis focused on children and adolescents found that heat exposure was associated with an increased risk of hospital visits or hospitalizations for mental health disorders, including depression and schizophrenia-related outcomes, highlighting early life vulnerability to climate-related psychiatric risk [10].

Despite this expanding epidemiological evidence, the biological mechanisms linking heat exposure to psychiatric outcomes remain poorly understood. This is a critical gap. Heat exposure directly challenges thermoregulation, a homeostatic function coordinated by central neural circuits that integrate thermal sensory input with autonomic, endocrine, cardiovascular, and behavioral responses [11,12,13]. Human heat stress activates heat-loss mechanisms, such as cutaneous vasodilation and sweating; however, these responses depend on hydration status, cardiovascular reserve, age, acclimatization, medication exposure, and environmental conditions, such as humidity and nighttime temperature [11]. Central thermoregulatory pathways involve the preoptic area of the hypothalamus and downstream autonomic circuits, placing heat regulation in close anatomical and functional proximity to the systems involved in sleep, arousal, stress reactivity, energy balance, and affective regulation [12,13]. Thus, psychiatric vulnerability during heat exposure may emerge not only from discomfort or environmental adversity but also from the disruption of biological systems that are central to emotional and cognitive stability.

Several mechanistic pathways may mediate the relationship between heat exposure and mental health. Neuroinflammation is a leading candidate because inflammatory signaling within the central nervous system can modify neuronal function, synaptic plasticity, neurotransmission, and behavior [14]. Inflammatory pathways are also implicated in depression and other stress-related psychiatric disorders, with cytokine-neurotransmitter interactions providing a biologically plausible bridge between peripheral immune activation and mood, motivation, cognition, and fatigue [15]. Heat stress may also contribute to oxidative stress, a state characterized by an imbalance between reactive oxygen species and antioxidant defenses, with potential consequences for proteins, lipids, DNA, mitochondrial function, and neuronal integrity [16]. In severe heat illness, central nervous system injury has been linked to neuroinflammatory responses, blood–brain barrier dysfunction, cerebral edema, altered cerebral perfusion, and neuronal injury, although the extent to which milder or repeated heat exposure produces subclinical neuropsychiatric effects remains unclear [17]. Blood–brain barrier dysfunction may be particularly relevant because inflammatory and oxidative processes can compromise neurovascular integrity, potentially amplifying neuroinflammation and altering brain homeostasis [18].

Sleep disruption is another central biological pathway connecting heat exposure and psychiatric outcomes. Sleep is tightly coupled with thermoregulation, and elevated nighttime temperatures can interfere with heat dissipation, delay sleep onset, reduce sleep duration, and impair sleep quality [19,20]. Large-scale wearable-based evidence suggests that warmer nights shorten sleep globally, with larger effects among older adults, women, residents of lower-income countries, and individuals living in warmer regions [19]. A systematic review of ambient heat and sleep similarly concluded that higher indoor or outdoor temperatures are generally associated with degraded sleep quantity and quality, with limited evidence of rapid adaptation effects [20]. This pathway is highly relevant to psychiatry because sleep disturbance is both a symptom and a precipitating factor for multiple mental disorders, including depression, anxiety, bipolar disorder, psychosis, and suicidal behaviors. Therefore, heat-related sleep loss may act as a biological and behavioral amplifier, converting environmental thermal strain into affective instability, impaired cognitive control, irritability, impulsivity, and relapse vulnerability.

Individuals living with mental disorders may be especially vulnerable to heat-related biological stress. A systematic review of mental illness and extreme heat concluded that individuals with mental illness are at an increased risk of negative health effects during extreme heat events, likely because of the interaction of biological, behavioral, pharmacological, and social factors [21]. Psychotropic medications may further modify heat vulnerability through their effects on sweating, thirst perception, fluid balance, autonomic regulation, renal function, and central thermoregulation [22,23]. Antipsychotics, antidepressants, lithium, anticholinergic agents, sedatives, and medications used for comorbid medical conditions may all become clinically relevant during periods of extreme heat, particularly in older adults, people with severe mental illness, and individuals with limited access to cooling, hydration, or health care [21,22,23]. This makes heat exposure not only a population-level mental health concern but also a practical clinical issue for psychiatric prescribing, relapse prevention and climate-informed care.

Taken together, the current evidence suggests that heat exposure may influence mental health through multiple interacting mechanisms rather than a single pathway. Thermoregulatory strain, neuroinflammation, oxidative stress, blood–brain barrier dysfunction, hypothalamic–pituitary–adrenal axis activation, sleep and circadian disruption, neurotransmitter changes, dehydration, and psychopharmacological vulnerability may converge to increase psychiatric risk. However, these pathways are often discussed separately in climate medicine, neuroscience, sleep research, psychiatry, psychopharmacology, and public health, resulting in a fragmented multidisciplinary literature. To address this gap, the present narrative review was guided by five objectives: first, to synthesize the biological mechanisms linking acute and chronic heat exposure with climate-related psychiatric outcomes; second, to integrate evidence from climate medicine, neuroscience, psychiatry, sleep research, and psychopharmacology into a unified biological framework; third, to distinguish pathways supported by stronger human evidence from those supported mainly by experimental, preclinical, severe-spectrum, or indirect mechanistic evidence; fourth, to connect these mechanisms with major psychiatric outcomes and susceptible population groups; and fifth, to translate this synthesis into implications for future research, clinical practice, and climate adaptation in mental health care.

## 2. Narrative Review Approach

This study was designed as a narrative review. The purpose was not to answer a narrowly defined question through meta-analysis but to integrate evidence from several partially overlapping fields, including environmental physiology, climate medicine, neuroscience, psychoneuroimmunology, sleep medicine, psychopharmacology, epidemiology, and psychiatry. Narrative reviews are particularly useful when a topic is conceptually broad, mechanistically complex, and insufficiently addressed by a single study design or outcome category [24,25]. This approach was thus considered appropriate for synthesizing biological pathways linking heat exposure with psychiatric outcomes and identifying conceptual gaps and priorities for future research.

A literature search was conducted using PubMed/MEDLINE, Scopus, Web of Science, and Google Scholar. Search terms were combined using concepts related to exposure, mechanisms, and outcomes. Exposure-related terms included “heat exposure,” “ambient temperature,” “high temperature,” “heatwave,” “thermal stress,” “heat stress,” “hyperthermia,” and “climate change.” The mechanistic terms included “thermoregulation,” “hypothalamus,” “preoptic area,” “autonomic nervous system,” “cardiovascular strain,” “dehydration,” “neuroinflammation,” “cytokines,” “oxidative stress,” “mitochondria,” “blood–brain barrier,” “hypothalamic–pituitary–adrenal axis,” “cortisol,” “sleep,” “circadian rhythm,” “serotonin,” “dopamine,” “noradrenaline,” “GABA,” and “psychotropic medication.” Outcome-related terms included “mental health,” “psychiatric disorders,” “depression,” “anxiety,” “bipolar disorder,” “schizophrenia,” “psychosis,” “substance use,” “suicide,” “self-harm,” “psychiatric admission,” and “emergency department.”

The search covered literature available from database inception to June 2026. English-language peer-reviewed publications and major institutional reports were prioritized. No strict language restriction was applied; however, only one Spanish-language source was retained because of its direct relevance to psychopharmacology and medication safety in psychiatric practice. Because this was a narrative review rather than a systematic review, no formal PRISMA flow diagram was produced. However, to improve transparency, approximately 350 records were screened at the title, abstract, or source level, approximately 150 full-text articles, reports, or book chapters were assessed for relevance, and 103 sources were ultimately cited.

Priority was given to systematic reviews, meta-analyses, large population-based studies, mechanistic human studies, experimental physiology studies, and influential reviews on thermoregulation, neuroinflammation, sleep, stress biology, and psychopharmacology. Animal and cellular studies were considered when they provided mechanistic insights relevant to plausible biological pathways, particularly neuroinflammatory, oxidative, blood–brain barrier, and thermoregulatory mechanisms. Studies were not excluded solely on the basis of geography because the aim was to develop a biologically informed framework applicable across climatic and socioeconomic contexts. However, special attention was given to evidence relevant to vulnerable populations, low- and middle-income settings, and individuals with mental disorders.

Eligible sources included peer-reviewed original studies, systematic reviews, meta-analyses, major narrative or conceptual reviews, experimental physiology studies, mechanistic animal or cellular studies, and major institutional reports when they were directly relevant to heat exposure, biological mechanisms, psychiatric outcomes, vulnerable populations, psychopharmacological heat vulnerability, or climate-health adaptation. Sources were excluded when they were unrelated to heat exposure or mental health, focused exclusively on non-psychiatric outcomes without mechanistic relevance to the review, lacked sufficient scientific or institutional credibility, or did not contribute substantively to the biological, clinical, or public health synthesis.

Because this review integrates heterogeneous evidence, findings were interpreted according to the type and evidentiary role of the source. Human epidemiological and clinical studies were used primarily to characterize associations between heat exposure and psychiatric outcomes. Human experimental and physiological studies were used to support thermoregulatory, cardiovascular, autonomic, sleep-related, and stress-response mechanisms. Animal and cellular studies were used to support mechanistic plausibility, particularly for neuroinflammatory, oxidative, mitochondrial, and blood–brain barrier pathways when direct psychiatric evidence was unavailable. Evidence from heatstroke or severe hyperthermia was interpreted as severe-spectrum mechanistic evidence and was not treated as direct proof of psychiatric effects during ordinary ambient heat exposure. Therefore, the synthesis differentiates established human associations from plausible mechanisms supported mainly by experimental, preclinical, or indirect evidence. This distinction is examined in greater detail later in the manuscript, in Section 11, where the primary evidence base and narrative credibility supporting each biological pathway are explicitly discussed.

The synthesis was organized mechanistically rather than by psychiatric diagnosis alone. First, the review describes heat exposure as a biological stressor that affects thermoregulation, autonomic and cardiovascular function, immune signaling, oxidative balance, neurovascular integrity, stress-response systems, sleep, circadian rhythms, and neurotransmission. Second, it examines how these pathways may converge with psychiatric outcomes, including depression, anxiety, bipolar disorder, schizophrenia-spectrum disorders, substance-related outcomes, and suicide risk. Third, it discusses vulnerability modifiers, including age, severe mental illness, psychotropic medication exposure, comorbid medical illnesses, housing conditions, occupational exposure, and limited access to cooling. Finally, it proposes an integrative biological framework and future research agenda for climate-informed psychiatry and geopsychiatry.

As this was a narrative review, no formal meta-analysis, pooled effect estimate, or risk-of-bias scoring was performed. Instead, this review aimed to provide a critical and integrative synthesis of the current evidence, with an emphasis on biological plausibility, translational relevance, and gaps requiring future empirical testing.

## 3. Heat Exposure and Human Thermoregulation

### 3.1. Heat Exposure as a Homeostatic Challenge

Humans maintain an internal temperature within a relatively narrow range despite major fluctuations in external temperature, humidity, metabolic heat production, clothing, physical activity, hydration status, and cardiovascular demand [11]. Heat exposure becomes biologically consequential when environmental and internal heat loads exceed the body’s capacity to dissipate heat. Under these conditions, thermoregulatory systems must coordinate central sensing, autonomic output, cardiovascular redistribution, sweating, behavioral adaptation, and endocrine responses to maintain homeostasis [11,12,13]. From a psychiatric perspective, this is important because the same physiological systems that maintain thermal balance are closely connected to arousal, sleep, stress reactivity, interoception, fatigue, cognition, and emotion regulation.

Heat stress should be understood as more than just a subjective experience of discomfort. It is a systemic biological state that can alter the cardiovascular workload, hydration, autonomic tone, inflammatory signaling, sleep continuity, and neuroendocrine regulation [11,26,27,28,29,30]. These processes are not psychiatric mechanisms in isolation, but they create biological conditions under which psychiatric symptoms may emerge, worsen, or become more difficult to regulate. For example, heat-related dehydration, tachycardia, fatigue, poor sleep, and autonomic arousal may increase irritability, anxiety, cognitive inefficiency, emotional dysregulation, and vulnerability to relapse in individuals with pre-existing mental disorders.

The psychiatric relevance of heat exposure also depends on whether the exposure is acute, repeated, or chronic. Acute heat exposure, such as during a heatwave, may produce rapid physiological strain through dehydration, sleep disruption, autonomic activation, and medication-related vulnerability. Repeated or chronic heat exposure may contribute to the cumulative biological burden, particularly when nighttime temperatures remain elevated and recovery periods are shortened. This distinction is important because psychiatric outcomes may arise from both short-term destabilization and a longer-term allostatic load.

### 3.2. Central Thermoregulation and the Hypothalamus

Thermoregulation is coordinated by distributed neural systems; however, the preoptic area of the anterior hypothalamus plays a central integrative role [12,13,26,27]. Thermosensitive neurons in and around the preoptic-anterior hypothalamus receive information from central and peripheral thermal signals, including inputs from the skin, spinal, visceral, and core temperature-sensing pathways [13,27]. These signals are integrated with effector systems that regulate heat loss and conservation responses, including cutaneous vasodilation, sweating, shivering, brown adipose tissue thermogenesis, and behavioral thermoregulation [12,13,26,27].

This role of the hypothalamus is highly relevant to mental health because the hypothalamus is not an isolated thermostat. It is a regulatory hub connected to sleep–wake control, endocrine function, autonomic output, feeding, energy balance, circadian physiology, and stress response systems [12,13]. The preoptic area, in particular, has been implicated in the coordination of both sleep and body temperature regulation, supporting the idea that heat exposure may influence psychiatric outcomes partly through overlapping thermoregulatory and sleep-related neural circuits [12,13]. Thus, a warming environment may place sustained pressure on brain systems that participate in arousal, rest, stress, and affective regulation.

Central thermoregulation also provides a conceptual bridge between environmental exposure and subjective psychiatric experiences. Thermal signals are not only processed as autonomic information but are also associated with interoceptive awareness, discomfort, behavioral urgency, and motivational states. When heat exposure persists, especially in the context of poor sleep, dehydration, crowding, occupational strain, or limited access to cooling, thermoregulatory stress may become psychologically salient and biologically destabilizing. This is especially relevant for individuals with anxiety, mood instability, psychosis, cognitive impairment, or a reduced ability to initiate protective behaviors.

### 3.3. Autonomic and Cardiovascular Responses to Heat

The primary human heat-loss responses are sweating and increased skin blood flow, both of which require coordinated autonomic and cardiovascular adjustments [11,28,29]. During heat exposure, cutaneous vasodilation increases blood flow to the skin, facilitating heat transfer from the core of the body to the external environment. Sweating allows evaporative heat loss, but its effectiveness depends on humidity, air movement, clothing, hydration, and the ability to replace lost fluids and electrolytes [11]. When these compensatory mechanisms are insufficient, the core temperature may rise, cardiovascular strain increases, and the risk of heat-related illness becomes greater.

The cardiovascular system is central to heat dissipation because blood flow distributes heat from the internal tissues to the skin [28]. Heat stress can increase the heart rate and cardiac output, reduce the central blood volume, alter blood pressure regulation, and increase the cardiovascular workload [28,29,30]. A recent meta-analysis of more than 400 laboratory-based heat exposure studies showed that heat exposure is associated with increases in core temperature and heart rate and that cardiac responses vary substantially according to the heat exposure modality used in experimental settings [30]. These findings are important for climate-health research because laboratory models do not always reproduce real-world heatwave exposure, yet they clarify the physiological burden imposed by thermal stress in these conditions.

Although cardiovascular strain is usually discussed in relation to cardiovascular morbidity, it also has psychiatric relevance. Autonomic arousal, palpitations, dizziness, fatigue, weakness, and heat-related discomfort can overlap with or amplify anxiety symptoms. In individuals with panic vulnerability, trauma-related hyperarousal, somatic symptom burden, or severe mental illness, heat-induced bodily sensations may be misinterpreted as danger, contributing to distress or reducing behavioral control. In addition, dehydration and electrolyte imbalance may impair cognition, increase fatigue, alter medication tolerability, and worsen vulnerability to delirium or acute behavioral disturbance in medically fragile individuals.

### 3.4. Sweating, Hydration, and Behavioral Thermoregulation

Sweating is essential for evaporative cooling; however, it introduces a trade-off between heat dissipation and fluid-electrolyte balance [11]. When fluid losses are not replaced, dehydration can reduce plasma volume, increase cardiovascular strain, impair thermoregulatory efficiency, and contribute to fatigue, dizziness, headaches, and cognitive difficulties. These effects may be especially relevant for psychiatric populations because some individuals with mental disorders have reduced access to hydration, impaired self-care, altered thirst perception, substance use, homelessness, institutional exposure, and medication-related vulnerability.

Behavioral thermoregulation is also important. Humans respond to heat not only through autonomic physiology but also through behaviors such as seeking shade, reducing physical activity, increasing fluid intake, removing clothing layers, using fans or air conditioning, and changing their sleep arrangements [11,12]. These behaviors require perception, motivation, planning, mobility, resources, and social support to be successful. Psychiatric disorders can interfere with these adaptive responses. For example, depression may reduce motivation and self-care, psychosis may impair judgment, cognitive impairment may limit risk recognition, substance use may increase exposure, and social isolation may delay help-seeking. Thus, psychiatric vulnerability to heat is partly biological and partly behavioral, with each domain reinforcing the other.

The interaction between autonomic and behavioral thermoregulation is central to the climate-informed psychiatric framework. Heat exposure may become dangerous not only because physiological systems fail, but also because the behaviors needed to reduce exposure are unequally available. Access to cooling, safe housing, green spaces, hydration, healthcare, and social support can determine whether thermoregulatory strain remains manageable or progresses toward biological and psychiatric destabilization.

### 3.5. Acute Versus Chronic Heat Exposure

Acute and chronic heat exposure may influence mental health through distinct mechanisms. Acute exposure, such as a heatwave or a sudden rise in nighttime temperature, may produce rapid biological effects through autonomic activation, dehydration, sleep loss, medication intolerance, and increased emergency presentations [5,6,7,11,21,22,23]. These short-term pathways are likely to be especially relevant for acute anxiety, agitation, insomnia, mood destabilization, psychotic relapse, substance-related crises, and suicide risk.

Chronic or repeated heat exposure may have different effects. Persistent exposure to high ambient temperatures, especially when combined with elevated nighttime temperatures, poor housing, occupational heat, urban heat islands, or limited access to cooling, may reduce the opportunities for physiological recovery. Over time, repeated thermoregulatory strain may contribute to allostatic load, cumulative sleep disruption, chronic fatigue, inflammatory priming, and reduced psychosocial stress resilience. Although direct mechanistic evidence linking chronic heat exposure to long-term psychiatric biology remains limited, this pathway is plausible, given the known effects of heat stress on thermoregulation, cardiovascular strain, sleep, and stress biology [11,19,20,28,29,30].

This distinction has implications for research design. Short-term time-series and case-crossover studies are well-suited for identifying acute heat-related changes in psychiatric emergency visits, admissions, and suicide mortality. However, chronic mechanisms require longitudinal designs that integrate temperature exposure histories, nighttime heat, housing conditions, sleep measures, biomarkers, medication data, and repeated psychiatric assessments. Without this distinction, climate psychiatry risks treating heat as a single exposure, when its biological effects likely vary according to duration, timing, intensity, humidity, recovery opportunity, and individual vulnerability.

## 4. Neuroinflammation and Immune Activation

### 4.1. Heat Exposure, Systemic Inflammation, and Neuroimmune Signaling

Neuroinflammation is one of the most biologically plausible pathways linking heat exposure to psychiatric outcomes. In general, neuroinflammation refers to immune signaling within the central nervous system involving microglia, astrocytes, endothelial cells, perivascular immune cells, cytokines, chemokines, complement, and peripheral-to-central immune communication [14]. This process is not intrinsically pathological; inflammatory signaling participates in host defense, tissue repair, synaptic remodeling, and homeostatic regulation. However, excessive, prolonged, or poorly resolved neuroinflammatory activation can alter neuronal excitability, neurotransmission, synaptic plasticity, neurovascular integrity, and behavior [14,15].

Heat exposure may contribute to neuroimmune activation via several overlapping routes. Severe hyperthermia and heatstroke are associated with systemic inflammatory responses, endothelial activation, coagulation abnormalities, gut barrier dysfunction, and central nervous system involvement [17,31,32]. In heatstroke, inflammatory mediators such as interleukin-1β, interleukin-6, tumor necrosis factor-α, interferon-γ, soluble tumor necrosis factor receptors, and other cytokines have been implicated in systemic inflammatory response syndrome-like physiology [32]. Although heatstroke represents the severe end of the heat illness spectrum, it provides mechanistic insights into how thermal stress can trigger immune activation, neurovascular injury, and brain dysfunction. The key translational question for psychiatry is whether milder but repeated heat exposure induces subclinical immune and neuroimmune changes sufficient to influence mood, cognition, sleep, arousal, or behavioral regulation.

Several mechanisms may connect peripheral heat-related inflammation to the brain. Hyperthermia can impair gastrointestinal barrier integrity, facilitating bacterial translocation and exposure to endotoxins, such as lipopolysaccharides, which can amplify systemic cytokine release [31,32]. Circulating inflammatory mediators may then communicate with the brain through humoral routes, circumventricular organs, vagal pathways, endothelial activation, and blood–brain barrier disruption [14,31,32]. Once central immune signaling is activated, microglia and astrocytes may shift toward reactive phenotypes that influence synaptic signaling, neuronal metabolism, and stress-related behaviors [14,15]. This provides a plausible biological link between environmental heat exposure and psychiatric symptoms such as fatigue, irritability, low mood, anxiety-like arousal, cognitive slowing, and reduced motivational capacity.

### 4.2. Microglial Activation and Heat-Related Brain Vulnerability

Microglia are the central mediators of neuroimmune responses. Under physiological conditions, they monitor the neural microenvironment and contribute to synaptic pruning, immune surveillance, and tissue repair. However, under inflammatory or metabolic stress, microglia can produce cytokines, reactive oxygen species, nitric oxide, prostaglandins, and other mediators that alter neuronal function [14]. Heat-related microglial activation has been described in experimental models of heat stress and heat-induced neural injury, supporting the idea that thermal stress can directly or indirectly engage the neuroimmune pathways.

Li et al. showed that heat stress induced inflammatory responses in BV-2 microglial cells, including increased release of interleukin-1β, interleukin-6, and tumor necrosis factor-α, increased expression of microglial activation markers, and activation of nuclear factor-κB signaling [33]. The same study found that microRNA-155 promoted heat stress-induced inflammation by targeting liver X receptor α, suggesting a molecular pathway through which heat exposure may amplify microglial inflammatory signaling [33]. Although this evidence comes from an experimental cellular model rather than human psychiatric populations, it strengthens the biological plausibility by showing that heat stress can activate inflammatory signaling within microglial systems relevant to brain function.

The psychiatric relevance of microglial activation is supported by extensive psychoneuroimmunology research. Inflammatory signaling can induce “sickness behavior,” a coordinated set of behavioral changes that includes fatigue, social withdrawal, reduced activity, sleep changes, anhedonia-like behavior, and cognitive inefficiency [34]. These adaptive responses during acute infection or injury phenomenologically overlap with the symptoms observed in depressive and stress-related disorders. In depression, inflammatory pathways have been linked to altered monoamine metabolism, glutamatergic signaling, hypothalamic–pituitary–adrenal axis function, neuroplasticity, and reward processing [15,34,35]. Therefore, heat-induced neuroimmune activation could represent a route through which environmental thermal stress contributes to psychiatric symptom expression, particularly in individuals with pre-existing inflammatory vulnerability or recurrent mood disorders.

### 4.3. Inflammation as a Transdiagnostic Psychiatric Pathway

Inflammation should not be conceptualized as being specific to one psychiatric diagnosis. Instead, it may function as a transdiagnostic biological pathway influencing affective, cognitive, somatic, and behavioral domains of emotion regulation. Elevated inflammatory activity has been most extensively studied in depression; however, immune dysregulation has also been implicated in anxiety disorders, bipolar disorder, schizophrenia-spectrum disorders, neurocognitive impairment, and stress-related conditions [14,15,35]. The clinical manifestations of inflammation may vary depending on genetic susceptibility, developmental timing, stress exposure, sleep disruption, metabolic status, medication use, and comorbidities.

This transdiagnostic view is particularly useful in climate-related psychiatry. Heat exposure does not map neatly onto any single psychiatric disorder. Epidemiological studies have suggested associations with mental health-related emergency visits, hospitalizations, psychiatric admissions, psychological distress, suicide mortality, and exacerbation of severe mental illness [5,6,7,8,9,10,21]. Neuroinflammation may provide a biological explanation for this diagnostic heterogeneity. Rather than causing a specific syndrome, heat-related inflammatory signaling may lower the threshold for symptom worsening across multiple disorders by increasing fatigue, arousal, irritability, cognitive inefficiency, sleep disturbance, and stress.

Direct human studies linking ambient heat exposure, neuroinflammatory biomarkers, and psychiatric outcomes remain scarce. Therefore, neuroinflammation should be interpreted as a biologically plausible and empirically testable pathway rather than as a proven causal mechanism.

## 5. Oxidative Stress, Mitochondrial Dysfunction, and Cellular Injury

### 5.1. Heat Stress and Oxidative Imbalance

Oxidative stress is another central pathway through which heat exposure affects brain function. Oxidative stress occurs when reactive oxygen and nitrogen species exceed the antioxidant defense capacity, resulting in damage to proteins, lipids, nucleic acids, mitochondria, and cellular membranes [16]. Reactive species also act as signaling molecules under physiological conditions; however, excessive or poorly regulated oxidative activity can impair cellular function and promote inflammation, endothelial dysfunction, and cell death [16].

Heat stress can increase reactive oxygen species production through multiple mechanisms, including mitochondrial electron transport chain dysfunction, protein denaturation, altered calcium homeostasis, membrane instability, inflammation, ischemia–reperfusion-like processes, and activation of enzymatic sources of oxidants [36]. In the context of severe heat illness, oxidative stress has been implicated in neuronal injury, endothelial damage, and multi-organ dysfunction [17,31,32,36]. Importantly, oxidative stress and inflammation are mutually reinforcing processes. Cytokine signaling can increase reactive oxygen species, whereas oxidative stress can activate inflammatory pathways, such as nuclear factor-κB, inflammasomes, and endothelial adhesion cascades [16,32,36].

This interaction is highly relevant to mental health. Oxidative and nitrosative stress have been implicated in depression and mood disorders, with proposed effects on neuroplasticity, mitochondrial function, inflammatory tone, monoamine metabolism, glutamatergic signaling, lipid peroxidation, and cellular aging [37,38]. Although psychiatric disorders cannot be reduced to oxidative damage, redox imbalance may contribute to symptom persistence, cognitive dysfunction, fatigue, treatment resistance, and neuroprogressive illness models. Accordingly, heat exposure could intensify pre-existing oxidative vulnerability, especially when combined with sleep loss, dehydration, inflammation, medication exposure, substance use, metabolic disease, or poor nutritional status.

### 5.2. Mitochondrial Dysfunction as a Convergent Mechanism

Mitochondria are central to the biological effects of heat exposure because they regulate energy production, redox balance, calcium signaling, apoptosis, innate immune signaling, and cellular adaptation. Heat stress can disrupt the mitochondrial membrane potential, impair oxidative phosphorylation, increase mitochondrial reactive oxygen species, damage mitochondrial DNA, alter mitochondrial dynamics, and activate cell death pathways [36,39]. In immune cells, mitochondrial injury may amplify inflammation by releasing mitochondrial DNA, cytochrome c, and other damage-associated molecular patterns that activate innate immune pathways [39]. This creates a feed-forward loop in which heat-induced mitochondrial dysfunction promotes oxidative stress, inflammation, endothelial dysfunction and tissue injury.

Although much of the heat-stress mitochondrial literature focuses on systemic illness, leukocyte dysfunction, organ failure, and exercise physiology, the implications for psychiatric biology are significant. The brain is highly energy-dependent, and neuronal signaling, synaptic plasticity, glial function, and neurotransmitter cycling require tightly regulated mitochondrial activities. Even modest disturbances in mitochondrial function may influence fatigue, cognition, emotional regulation and stress resilience. Psychiatric research has increasingly implicated mitochondrial dysfunction in major depression, bipolar disorder, schizophrenia, and stress-related conditions, although the causal pathways remain complex and heterogeneous [37,38].

Heat-related mitochondrial dysfunction may be particularly relevant under conditions of repeated or chronic exposure. Acute thermal stress may induce transient mitochondrial and redox changes that resolve with cooling and recovery. However, repeated heat exposure, particularly when accompanied by inadequate nighttime recovery, chronic sleep restriction, dehydration, occupational heat strain, or systemic inflammation, may contribute to cumulative energetic stress. This provides a mechanistic basis for investigating whether chronic heat exposure contributes to psychiatric vulnerability via allostatic and mitochondrial pathways.

### 5.3. Heat Shock Proteins and Cellular Adaptation

Heat shock proteins are molecular chaperones that help maintain protein folding, prevent aggregation, support cellular repair, and promote survival under thermal and other stressful conditions [32,36]. Heat shock protein responses are part of an adaptive cellular defense system that allows organisms to tolerate transient temperature elevations. Therefore, not all biological responses to heat are harmful. Some responses are protective, especially when heat exposure is moderate, short-lived, and followed by adequate recovery periods. This adaptive response provides a biological basis for distinguishing potentially protective short-term heat adaptation from harmful heat stress, particularly when exposure is mild and accompanied by adequate cooling, hydration, and physiological recovery [11,32,36].

However, severe or prolonged heat exposure can overwhelm the adaptive chaperone systems. Protein misfolding, mitochondrial injury, oxidative stress, and inflammatory activation may exceed the compensatory capacity [31,32,36]. In heatstroke, this breakdown is associated with a systemic inflammatory response, coagulation abnormalities, endothelial dysfunction, and central nervous system involvement [17,31,32]. In psychiatry, the concept of adaptive versus maladaptive heat response is important because it helps explain why the same environmental exposure may produce different outcomes depending on acclimatization, health status, medication use, sleep, hydration, age, socioeconomic resources, and pre-existing psychiatric vulnerability.

This distinction is also relevant for future biomarker studies. Heat shock proteins, oxidative stress markers, inflammatory cytokines, mitochondrial DNA, cortisol, sleep metrics, and autonomic measures may help to characterize individual responses to heat exposure. Accordingly, climate-informed psychiatric biology should move beyond measuring ambient temperature alone and toward integrated exposure-response models that include physiological adaptation, recovery, and vulnerability.

## 6. Blood–Brain Barrier Dysfunction and Neurovascular Pathways

### 6.1. Heat and Blood–Brain Barrier Integrity

The blood–brain barrier is a specialized neurovascular interface composed primarily of brain microvascular endothelial cells, tight junctions, pericytes, astrocytic end-feet, basement membranes, and associated immune and neuronal elements. It regulates molecular exchange between the peripheral circulation and central nervous system, thereby maintaining brain homeostasis and protecting neural tissue from toxins, pathogens, and excessive inflammatory signaling [18]. Blood–brain barrier dysfunction can amplify neuroinflammation by allowing circulating cytokines, immune cells, microbial products, and other inflammatory mediators to influence the central nervous system [18].

Heat exposure may compromise blood–brain barrier integrity through direct thermal effects, endothelial injury, oxidative stress, inflammatory mediators, and altered cerebral blood flow [17,18,31,40,41]. Kiyatkin and Sharma experimentally showed that blood–brain barrier permeability depends on brain temperature, supporting the concept that hyperthermia can alter barrier function [40]. Yamaguchi et al. further demonstrated that heat stress affects blood–brain barrier integrity in vivo and in vitro, including vascular leakage in heatstroke model mice, reduced trans-endothelial electrical resistance in induced pluripotent stem cell-derived brain microvascular endothelial cells, and reduced claudin-5 expression under heat stress [41]. These findings are particularly important because they directly link heat exposure to neurovascular barrier biology.

### 6.2. Neurovascular Dysfunction and Psychiatric Relevance

Blood–brain barrier disruption may contribute to psychiatric vulnerability by increasing central exposure to peripheral inflammatory signals and altering the neurovascular environment required for normal neuronal and glial function [18]. Neurovascular dysfunction influences cerebral perfusion, endothelial signaling, oxidative stress, synaptic regulation, and immune cell trafficking. In severe heat illness, neurological dysfunction may include altered consciousness and cognitive impairment; however, the psychiatric relevance extends beyond acute neurological syndromes [17,31,32]. Subclinical neurovascular changes could plausibly influence cognition, fatigue, irritability, affective instability, and stress sensitivity, although this remains an area that requires direct human studies.

The blood–brain barrier pathway also helps integrate several mechanisms that have already been discussed. Heat-related dehydration and cardiovascular strain may affect cerebral perfusion; oxidative stress may damage endothelial cells and tight junctions; systemic inflammation may activate endothelial signaling; and gut-derived endotoxin exposure may amplify cytokine responses [18,31,32,40,41]. These processes may converge at the neurovascular unit, making it a key interface between environmental heat and the brain.

### 6.3. Research Implications

Direct human psychiatric evidence on heat-related blood–brain barrier dysfunction remains limited. Future studies should examine whether real-world heat exposure is associated with markers of neurovascular stress, such as inflammatory biomarkers, endothelial activation markers, blood–brain barrier permeability indicators, neuroimaging measures, or cerebrospinal fluid changes in selected clinical contexts. Such studies would be particularly valuable in populations with severe mental illness, older adults, people taking thermoregulation-impairing medications, and individuals exposed to recurrently high nighttime temperatures.

## 7. Hypothalamic–Pituitary–Adrenal Axis and Stress Biology

### 7.1. Heat Exposure as a Physiological Stressor

Heat exposure can be conceptualized as a physiological stressor because it challenges homeostasis and requires coordinated neuroendocrine, autonomic, cardiovascular, immune, and behavioral adaptations [11,42]. The stress system is organized around interacting central and peripheral components, including corticotropin-releasing hormone, arginine vasopressin, adrenocorticotropic hormone, glucocorticoids, the sympathetic nervous system, and immune-inflammatory mediators [42]. These systems are adaptive when activated transiently, allowing organisms to respond to environmental demands. However, repeated, excessive, or poorly resolved activation may contribute to biological wear and tear, particularly when recovery is incomplete [43,44,45].

The hypothalamus has dual relevance in the context of heat. First, it coordinates thermoregulatory responses through the preoptic and downstream autonomic pathways [12,13,26,27]. Second, it participates in stress response regulation through hypothalamic–pituitary–adrenal axis signaling [42]. These functions are not isolated from each other. Thermal stress, autonomic arousal, dehydration, sleep loss, and inflammatory activation converge on the neuroendocrine stress systems. This convergence provides a plausible mechanism through which environmental heat exposure may influence psychiatric vulnerability, especially when heat exposure occurs repeatedly, during the night, or in individuals with reduced physiological reserves.

The relationship between heat exposure and activation of the hypothalamic–pituitary–adrenal axis is likely to be complex. Acute heat stress may increase physiological arousal and activate stress response pathways, whereas repeated exposure may produce adaptation, blunted responses, or dysregulated reactivity, depending on exposure intensity, duration, acclimatization, age, health status, and comorbid stressors [11,42]. This complexity is clinically important because psychiatric symptoms may arise not only from excessive activation but also from dysregulated stress responsivity. For example, fatigue, emotional lability, irritability, anxiety, cognitive inefficiency, and sleep disturbance may reflect different configurations of autonomic, endocrine, inflammatory, and circadian disruption, rather than a single uniform stress response.

### 7.2. Cortisol, Allostatic Load, and Psychiatric Vulnerability

Allostasis refers to the process by which physiological systems adapt to changing demands, whereas allostatic load refers to the cumulative biological cost of repeated or chronic adaptation [43,44]. Heat exposure is highly compatible with this framework. During acute thermal stress, autonomic and endocrine responses help to preserve temperature regulation, hydration, cardiovascular stability, and behavioral adaptation. However, when heat exposure is repeated, prolonged, or combined with insufficient nighttime recovery, the same adaptive systems may contribute to a cumulative physiological burden [11,19,20,43,44,45].

Allostatic load is not limited to cortisol levels. It involves the interaction of neuroendocrine, immune, metabolic, cardiovascular, and autonomic systems [43,44,45]. This is particularly relevant for climate-related psychiatric outcomes because heat exposure may simultaneously affect several of these domains: cardiovascular strain, dehydration, sleep disruption, inflammatory signaling, oxidative stress, neurovascular integrity, and medication tolerance [11,14,15,16,17,18,19,20,31]. In this sense, heat should not be treated only as an external exposure variable but also as a repeated biological demand that can interact with pre-existing psychiatric and medical vulnerability.

The allostatic framework also helps explain why psychiatric risk may be greater in socially and clinically vulnerable populations than in other populations. Individuals with severe mental illness, older adults, people with chronic medical illnesses, people living in poor-quality housing, outdoor workers, and those with limited access to cooling may experience heat as a repeated stressor without adequate recovery. Over time, this may increase vulnerability through cumulative sleep loss, fatigue, autonomic strain, inflammation, and impaired coping capacities. Although direct longitudinal studies linking heat exposure, allostatic load biomarkers, and psychiatric outcomes remain scarce, this pathway is biologically plausible and should be prioritized in future studies.

### 7.3. Interaction Between Biological Heat Stress and Psychological Climate Stress

Heat exposure is also associated with broader psychological stress related to climate change. Climate change can influence mental health through direct exposure to extreme weather, indirect socioeconomic and ecological disruption, and anticipatory distress regarding future environmental threats [3,4]. Therefore, heat operates at two levels. At one level, it is a direct biological stressor that acts on thermoregulation, sleep, autonomic function, and inflammatory pathways. At another level, it is a visible and increasingly frequent reminder of climate instability, potentially contributing to worry, perceived threats, helplessness, and community-level stress.

This dual role is important in psychiatric biology. Psychological stress can influence sleep, hypothalamic–pituitary–adrenal axis activity, autonomic regulation, inflammation, and behavior [42,43,44,45]. These pathways may reinforce each other when psychological climate stress occurs alongside heat exposure. For example, a heatwave may worsen sleep and physiological arousal while increasing anxiety about health, finances, work, electricity access, caregiving, or future climate risks. In vulnerable individuals, this combination may reduce resilience and increase the likelihood of exacerbating symptoms.

An integrative model should avoid separating “biological” and “psychological” heat effects too sharply. Heat exposure is biologically embodied, but its effects are shaped by perceptions, context, resources, social support, and meaning. This is especially relevant to geopsychiatry, which emphasizes that mental health emerges from the interaction between place, climate, social conditions and biological vulnerability.

## 8. Sleep Disruption as a Biological Mediator

### 8.1. Nighttime Heat and Sleep Architecture

Sleep is one of the most important biological mediators linking heat exposure to psychiatric outcomes. Human sleep is closely connected to thermoregulation: sleep onset is facilitated by heat dissipation, distal vasodilation, and a decline in core body temperature, whereas sleep stages are accompanied by distinct thermoregulatory patterns [46,47]. Environmental heat can interfere with these processes by limiting heat loss, increasing discomfort, increasing wakefulness, and altering the sleep architecture [20,46,47].

The effects of the thermal environment on sleep have been described in both laboratory and real-world contexts. Okamoto-Mizuno and Mizuno reported that in real-life situations where bedding and clothing are used, heat exposure increases wakefulness and decreases slow-wave and rapid eye movement sleep [46]. Harding et al. further emphasized that sleep and thermoregulation are deeply linked, with sleep onset and non-rapid eye movement sleep occurring in close relationship with body and brain cooling [47]. These findings provide mechanistic support for the idea that elevated nighttime temperatures may impair sleep not only through subjective discomfort but also through the disruption of the biological conditions required for stable sleep.

Recent climate-health evidence supports this pathway. Large-scale wearable data indicate that rising nighttime temperatures shorten human sleep globally, primarily by delaying sleep onset, with larger effects in older adults, women, residents of lower-income countries, and individuals living in warmer regions [19]. A systematic review of ambient heat and sleep concluded that higher outdoor and indoor temperatures are generally associated with degraded sleep quantity and quality, especially during hotter periods and among vulnerable populations [20]. Collectively, these findings suggest that nighttime heat may be a major pathway through which climate change affects mental health.

### 8.2. Sleep Loss and Psychiatric Outcomes

Sleep disturbance is not merely a nonspecific symptom of psychiatric disorders; it is increasingly understood as a contributing and bidirectional factor in mental health. Freeman et al. reviewed evidence that disrupted sleep may play a causal or maintaining role across major psychiatric conditions, and that improving sleep can reduce other mental health symptoms [48]. Hertenstein et al. similarly showed in a systematic review and meta-analysis that cognitive behavioral therapy for insomnia improves insomnia severity and mental health outcomes among patients with mental disorders and comorbid insomnia [49]. These findings support the clinical relevance of sleep as an intervention target rather than as a consequence of psychopathology.

Heat-related sleep disruption may influence psychiatric outcomes via several mechanisms. Sleep loss can increase emotional reactivity, reduce cognitive control, impair reward processing, increase pain sensitivity, worsen fatigue, and alter stress response and immune pathways. In the context of heat exposure, these effects may be amplified by dehydration, autonomic arousal, nocturnal discomfort, reduced recovery, and poor daytime performance. This may reflect a biological cascade in which nighttime heat produces sleep loss, sleep loss reduces emotional and cognitive regulation, and reduced regulation increases vulnerability to anxiety, depressive symptoms, irritability, impulsivity, and relapse.

This pathway is likely to be particularly important for individuals with pre-existing psychiatric disorders. Insomnia and sleep fragmentation may worsen depression and anxiety symptoms, destabilize bipolar disorder, increase psychotic experiences, impair substance use recovery, and reduce adherence to daily routines. In people living with severe mental illness, heat-related sleep disruption may be combined with medication-related thermoregulatory vulnerability, social isolation, comorbid medical illnesses, and limited access to cooling. Thus, sleep is a central point of convergence between environmental heat exposure and psychiatric clinical risk.

### 8.3. Heat, Circadian Rhythms, and Mood Regulation

Circadian rhythm disruption provides an additional pathway linking heat exposure to mental health. Body temperature follows a circadian rhythm, and the sleep–wake cycle is closely aligned with this rhythm [46,47]. When nighttime temperatures remain elevated, the normal decline in body temperature may be blunted or delayed, potentially interfering with sleep timing, consolidation, and circadian stability [19,20,46,47]. This is particularly relevant because climate change is associated not only with higher daytime temperatures but also with elevated nighttime temperatures, reducing opportunities for physiological recovery.

Mood disorders are particularly sensitive to circadian disruption. Sleep and circadian rhythm disturbances are well described in bipolar disorder and may contribute to relapse, affective instability, and impaired function [50,51]. Harvey emphasized that sleep disturbance in bipolar disorder affects the quality of life, relapse risk, and affective regulation [50]. Takaesu reviewed evidence that sleep–wake and circadian rhythm abnormalities are widely observed in bipolar disorder, including irregular sleep–wake rhythms and disturbances in mood states [51]. These findings are important for heat-related psychiatry because elevated nighttime temperatures may destabilize sleep and circadian timing in the domains that are clinically relevant for bipolar and other mood disorders.

Circadian disruption may also interact with inflammatory and stress responses. Sleep loss can increase inflammatory activity and alter hypothalamic–pituitary–adrenal axis regulation, whereas stress and inflammation can further impair sleep [14,15,34,35,42]. Therefore, heat-related sleep disruption may act as an independent biological pathway and an amplifier of other mechanisms described in this review. Thus, nighttime heat is a particularly important exposure metric for future studies. Psychiatric heat research should move beyond daily maximum temperature and incorporate nighttime minimum temperature, indoor thermal conditions, sleep duration, sleep timing, circadian regularity, and recovery periods.

## 9. Neurotransmitter Systems and Behavioral Regulation

### 9.1. Heat Exposure and Neurochemical Regulation

Neurotransmitter systems provide a plausible but less directly established pathway linking heat exposure to psychiatric symptoms. Heat exposure affects physiological systems that are regulated by or interact with central monoaminergic and inhibitory neurotransmission, including arousal, motivation, fatigue, stress response, mood, sleep–wake regulation, autonomic function, and behavioral control [52,53,54,55]. These domains are central to psychiatric disorders, suggesting that heat may alter neurochemical states that modulate symptom expression and clinical vulnerability.

Evidence linking heat, neurotransmitters, and behavior comes primarily from exercise physiology, fatigue research, thermoregulation studies, animal models, and experimental heat stress paradigms rather than psychiatric cohorts exposed to real-world climate-related heat [52,53,54,55]. Nevertheless, these studies indicate that heat stress can influence neurochemical and neuroendocrine systems involved in mood, cognition, fatigue, and arousal. Therefore, neurotransmission should be considered part of a broader multi-system model rather than an isolated explanatory pathway [52].

The clearest translational implication is that heat exposure may shift brain-body regulation toward heightened arousal, fatigue, reduced cognitive efficiency, irritability, and impaired behavioral control. These effects may be mediated through the interaction of serotonergic, dopaminergic, noradrenergic, GABAergic, glutamatergic, inflammatory, sleep-related, and endocrine pathways [15,42,52,53,54,55]. In clinical psychiatry, such shifts may be particularly relevant for patients with mood instability, anxiety sensitivity, psychotic vulnerability, substance use, insomnia, cognitive impairment, or reduced capacity for adaptive behavior during heat exposure.

### 9.2. Serotonergic Pathways

Serotonin is relevant to thermoregulation and mental health. It is involved in mood regulation, sleep, appetite, fatigue, impulsivity, aggression, and behavioral inhibition, and participates in central thermoregulatory processes and responses to physical stress [52,53,54]. Exercise and heat-stress research has long examined the “central fatigue” hypothesis, in which serotonergic activity, particularly in relation to dopaminergic tone, is proposed to influence fatigue, motivation, and perceived effort [53,54]. Although this literature does not map directly onto climate-related psychiatric outcomes, it provides a biologically plausible link between heat exposure, fatigue, affective states, and behavioral regulation.

Meeusen and colleagues argued that the interaction between serotonin, dopamine, and noradrenaline may be more informative than serotonin alone for understanding central fatigue and performance under heat stress [53,54]. This is relevant for mental health because fatigue, reduced motivation, cognitive inefficiency, and emotional dysregulation are common, transdiagnostic symptoms. When heat exposure disrupts sleep, increases cardiovascular strain, and activates stress systems, serotonergic pathways may contribute to subjective fatigue, mood changes, irritability, and reduced coping capacity of the individual.

Serotonergic pathways are clinically relevant because many antidepressants and other psychotropic medications act on serotonin signaling. During heat exposure, serotonergic medications may interact with thermoregulation, sweating, hydration, and rare but severe drug-induced hyperthermic syndromes [22,23,56,57,58,59]. This does not imply that antidepressants should be avoided during hot weather; rather, it indicates that medication effects should be considered within individualized heat-risk planning, especially in patients who are older, have comorbid medical illnesses, are on polypharmacy, have dehydration risk, or have limited access to cooling.

### 9.3. Dopaminergic Pathways

Dopamine is central to reward processing, motivation, motor activity, salience attribution, psychotic biology, and thermoregulatory pharmacology. In the context of heat exposure, dopaminergic pathways are relevant in two main ways. First, dopamine interacts with serotonin and noradrenaline in central fatigue and performance regulation during heat stress [53,54]. A lower capacity to sustain motivation, effort, and cognitive control under heat stress may be partly related to altered monoaminergic balance, although direct psychiatric evidence remains limited.

Second, dopamine is central to the pharmacological treatment of psychosis and bipolar disorder. Antipsychotic medications, which commonly act through dopamine D2 receptor antagonism or partial agonism, may influence thermoregulation and heat vulnerability [22,23,56,57]. This is especially important because schizophrenia-spectrum disorders and severe mental illnesses are consistently identified as risk factors during extreme heat events [21,22,23]. Dopaminergic blockade may interact with hypothalamic thermoregulatory pathways, sedation, reduced behavioral responsiveness, anticholinergic burden, and impaired self-care, potentially increasing the vulnerability to heat-related illnesses.

Dopaminergic pathways may also be relevant to human behaviors during heat exposure. Heat-related fatigue, sleep loss, irritability, and reduced executive function may impair decision-making and adaptive protective behaviors. In people with psychosis, mania, substance use, or cognitive impairment, this may reduce the likelihood of seeking shade, hydrating, avoiding exertion, modifying medication storage, or contacting healthcare services. Therefore, dopaminergic mechanisms should be understood not only as neurochemical pathways but also as part of the clinical ecology of severe mental illness during extreme heat.

### 9.4. Noradrenergic and Sympathetic Arousal Pathways

Noradrenergic signaling is central to arousal, vigilance, stress response, attention, autonomic regulation, and threat-processing. Heat exposure activates autonomic and cardiovascular responses, including increased heart rate, cutaneous vasodilation, sweating, and blood flow redistribution [11,28,29,30]. These responses are adaptive, but they can also produce bodily sensations that overlap with anxiety symptoms, including palpitations, dizziness, sweating, breathlessness, weakness, and agitation.

McMorris et al. examined heat stress in relation to plasma adrenaline, noradrenaline, 5-hydroxytryptamine, cortisol, mood state, and cognitive performance, finding that heat stress was associated with deterioration in a central executive task and changes in physiological and neurochemical markers [55]. Although peripheral plasma measures do not directly reflect central neurotransmission, this study supports the broader idea that heat stress can alter neuroendocrine and catecholaminergic states relevant to cognition and mood regulation. In psychiatric populations, these changes may be clinically meaningful when they occur alongside sleep loss, dehydration, or pre-existing anxiety sensitivity.

Noradrenergic arousal may be particularly relevant to anxiety, trauma-related symptoms, irritability, agitation, and insomnia. Heat exposure can make the body feel activated and uncomfortable; for individuals prone to panic or somatic misinterpretation, this may increase their distress. In addition, chronic heat exposure may reduce recovery and increase baseline arousal, contributing to the allostatic burden [42,43,44,45]. These pathways require direct study in psychiatric cohorts; however, they are biologically plausible and clinically recognizable.

### 9.5. GABAergic and Glutamatergic Balance

The GABAergic and glutamatergic systems regulate the inhibitory-excitatory balance, cortical excitability, anxiety, sleep, seizure threshold, cognitive processing, and stress adaptation. Heat exposure may indirectly influence this balance through sleep disruption, inflammation, oxidative stress, neuroendocrine activation, and altered neuronal excitability [14,15,16,31,46,47,48,49]. Severe hyperthermia and heatstroke can produce neurological dysfunction, including altered consciousness and neuronal injury; however, the psychiatric relevance of milder heat-related changes in excitatory-inhibitory balance remains unclear [17,31].

The main psychiatric implication is that heat exposure may reduce neurobehavioral stability by combining destabilizing inputs, including poor sleep, autonomic arousal, fatigue, dehydration, inflammation, oxidative stress, and medication-related vulnerability. In individuals with anxiety disorders, bipolar disorder, psychosis, epilepsy comorbidity, substance use, or sedative medication exposure, these interacting processes may influence arousal, inhibition, cognition, and behavioral regulation. Direct evidence in psychiatric populations remains limited, so excitatory-inhibitory balance should be treated as a research gap rather than an established heat-related psychiatric mechanism.

## 10. Psychopharmacology and Heat Vulnerability

### 10.1. Why Psychotropic Medications Matter During Heat Exposure

Psychopharmacology is one of the most clinically actionable pathways linking heat exposure to mental health. Many individuals at increased psychiatric risk during heat events are also prescribed medications that may influence thermoregulation, hydration, sweating, sedation, cognition, renal function, cardiovascular responses, and behavioral adaptation [22,23,56,57,58,59,60]. This does not imply that psychotropic medications are inherently unsafe in hot weather. Rather, it suggests that heat exposure alters the physiological context in which these medications are used.

Several mechanisms are involved. Medications may impair central thermoregulation, reduce sweating, alter thirst perception, increase sedation, impair cognition, contribute to orthostatic hypotension, modify cardiovascular responses, increase dehydration risk, or become toxic when the fluid balance and renal function are compromised [22,23,56,57,58,59,60]. These mechanisms are especially important among older adults, individuals with severe mental illness, individuals with medical comorbidities, individuals taking multiple medications, and individuals with limited access to cooling, hydration, or clinical monitoring.

Population-level evidence supports these clinical concerns. Martin-Latry et al. examined psychotropic drug use and heat-related hospitalizations during a heatwave period and found that psychotropic medications were associated with an increased risk of admission for heat-related pathology [56]. More recently, Wong et al. studied psychotropics and heat-related outcomes among people with mental health conditions and reported that psychotropics may modify the association between extreme temperatures and heat-related outcomes [22]. Xue et al. further emphasized that psychotropic prescriptions should be considered in the context of extreme heat because even short exposures may have clinically relevant consequences for vulnerable patients [23].

To refine the clinical boundaries of medication-related heat vulnerability, psychotropic risk should be considered across three interacting dimensions: medication intensity, regimen complexity, and patient vulnerability. Medication intensity includes dose, recent dose escalation, serum concentration where applicable, and the intrinsic thermoregulatory, anticholinergic, sedative, autonomic, renal, or cognitive effects of the agent. Regimen complexity includes polypharmacy, cumulative anticholinergic or sedative burden, combinations of psychotropics with overlapping adverse-effect profiles, and concomitant non-psychiatric medications that may affect hydration, blood pressure, renal perfusion, or heat dissipation. Patient vulnerability includes age, renal function, cardiovascular disease, cognitive impairment, substance use, dehydration risk, acute infection, diarrhea or vomiting, housing conditions, access to cooling, and capacity for symptom recognition and clinical monitoring. Thus, heat-related medication risk is not determined by drug class alone, but by the interaction between drug exposure, combined medication burden, comorbidities, and environmental heat conditions [22,23,56,57,58,59,60,61]. This three-dimensional clinical stratification is summarized in Supplementary Table S1.

### 10.2. Antipsychotics and Thermoregulatory Risk

Antipsychotics are among the most important medications for heat-related psychiatric vulnerability. They may increase the risk through several mechanisms, including effects on central thermoregulation, dopamine blockade, anticholinergic properties, sedation, reduced behavioral responsiveness, impaired sweating, orthostatic hypotension, and comorbid medical burden [22,23,56,57,58,59]. Individuals prescribed antipsychotics may also have schizophrenia-spectrum disorders, bipolar disorder, cognitive impairment, substance use, poverty, homelessness, institutional exposure, or social isolation, all of which may further increase heat vulnerability [21,22,23].

A more stratified interpretation is needed when considering antipsychotic-related heat vulnerability. First-generation antipsychotics may be particularly relevant because stronger dopamine D2 receptor blockade can interfere with central thermoregulatory pathways and may contribute to rigidity, reduced behavioral responsiveness, and rare hyperthermic syndromes. Some first-generation agents may also add anticholinergic, sedative, or hypotensive effects, depending on the specific drug and dose. Second-generation antipsychotics may also increase heat vulnerability, but the mechanisms may differ across agents and may include sedation, metabolic burden, orthostatic hypotension, anticholinergic effects, impaired self-care, and reduced behavioral thermoregulation. Therefore, heat-related antipsychotic risk should not be inferred from the first-generation versus second-generation distinction alone, but from the combined receptor profile, dose, anticholinergic burden, sedative burden, comorbid physical illness, hydration status, housing conditions, and access to cooling [22,23,56,57,58,59].

Antipsychotic-related heat risk should be distinguished from neuroleptic malignant syndrome, although both involve thermoregulation and dopaminergic pathways. Drug-induced hyperthermia reviews identify antipsychotics among medication classes capable of contributing to hyperthermic disorders; however, real-world heat vulnerability is often more gradual and multifactorial than classic neuroleptic malignant syndrome [59]. During heatwaves, the clinical issues are not only rare catastrophic hyperthermia but also dehydration, exhaustion, reduced self-care, delirium risk, worsened medical comorbidity, and increased emergency presentations.

For clinicians, this implies the need for anticipatory heat planning, rather than reactive crisis management. Patients taking antipsychotics may benefit from heat risk counseling, review of anticholinergic burden, monitoring of hydration and sedation, attention to living conditions, and coordination with caregivers or community services during heatwaves. Medication discontinuation or abrupt changes should not be promoted as a general strategy for managing heat, because the risk of relapse may be substantial. Instead, individualized clinical monitoring and prevention are essential.

### 10.3. Antidepressants, Anticholinergic Burden, and Sweating

Antidepressants may influence heat vulnerability through several pathways, depending on the class of medication. Selective serotonin reuptake inhibitors, serotonin-norepinephrine reuptake inhibitors, tricyclic antidepressants, and other antidepressants may affect sweating, serotonergic signaling, autonomic tone, sleep, gastrointestinal symptoms, and, in rare cases, drug-induced hyperthermic syndromes [22,23,57,58,59]. Tricyclic antidepressants and other medications with anticholinergic properties may be particularly relevant because reduced sweating can impair evaporative heat loss, whereas sedation or cognitive impairment may reduce protective behavior.

Antidepressant-related heat vulnerability also requires stratification. Selective serotonin reuptake inhibitors may be associated with altered sweating, serotonergic effects, gastrointestinal symptoms, and rare serotonin-toxicity-related hyperthermia, but individual SSRIs may differ in their pharmacokinetic profile, interaction potential, and tolerability. Serotonin-norepinephrine reuptake inhibitors may add noradrenergic and cardiovascular effects, such as changes in blood pressure, sweating, and autonomic tone. Tricyclic antidepressants are particularly relevant during heat exposure because their anticholinergic effects may reduce sweating and impair evaporative heat loss, while also increasing sedation, orthostatic hypotension, and cognitive impairment in vulnerable patients. Other antidepressants may influence heat risk through sedation, sleep effects, autonomic changes, or drug interactions. Thus, antidepressant-related heat risk should be assessed according to the specific agent, dose, anticholinergic burden, comorbidities, age, hydration status, and concomitant medications rather than by antidepressant class alone [22,23,57,58,59].

The relationship between antidepressants and heat exposure is complex. Some antidepressants may increase sweating, contributing to fluid loss, whereas anticholinergic medications may reduce sweating and impair cooling. Serotonergic drugs may also be clinically relevant in the differential diagnosis when hyperthermia occurs in the context of serotonin toxicity, although this is not the same as ordinary environmental heat illness [59]. Thus, the psychiatric heat-risk framework should avoid simplistic medication rankings and instead consider the mechanism, dose, patient age, comorbidity, polypharmacy, hydration status, renal function, and environmental exposure.

The recent systematic review and meta-analysis by Hospers et al. is important because it evaluated whether medications listed by health authorities as thermoregulation-impairing actually increase core temperature responses during heat stress [58]. This type of evidence is needed because clinical guidelines often include broad lists of medications despite limited direct experimental data. Future studies should examine specific psychotropic classes, combinations, and patient populations under realistic heat-exposure conditions.

### 10.4. Lithium, Dehydration, and Renal Vulnerability

Lithium requires special attention in heat-related psychiatric care because it has a narrow therapeutic index and is sensitive to fluid balance, renal function, sodium balance, and dehydration [60,61]. Heat exposure can increase sweating and fluid loss; when these are not replaced, dehydration may increase the risk of elevated lithium concentration or toxicity in susceptible individuals [23,57,60,61]. This is particularly relevant for patients with bipolar disorder, older adults, patients taking diuretics or renin-angiotensin system medications, and individuals with renal impairment or limited access to hydration.

Available evidence suggests that the relationship between environmental temperature, seasonality, and serum lithium concentrations is clinically relevant but not uniform [61]. In a large retrospective study from Sydney, Australia, Cheng et al. examined serum lithium concentrations in relation to recent maximum temperature and seasonal or monthly variation, based on the clinical concern that high environmental temperatures may contribute to dehydration, elevated plasma lithium concentration, and toxicity [61]. However, the study did not find a clinically meaningful increase in serum lithium concentrations during warmer periods [61]. Therefore, heat-related lithium vulnerability should not be interpreted as an inevitable seasonal increase in lithium levels. Rather, the highest-risk situations are those in which heat exposure coincides with dehydration, reduced oral intake, vomiting or diarrhea, renal impairment, older age, high or recently increased lithium dose, narrow therapeutic range, interacting medications, and limited access to serum lithium or renal-function monitoring [22,23,60,61].

Clinically, this supports anticipatory counseling, hydration monitoring, awareness of toxicity symptoms, and consideration of serum lithium and renal function checks in high-risk circumstances. The goal is not to discourage lithium use, which remains a highly effective treatment for bipolar disorder, but to integrate climate and heat exposure into routine medication safety planning.

### 10.5. Sedatives, Benzodiazepines, Substance Use, and Behavioral Risk

Sedatives, hypnotics, benzodiazepines, alcohol, and other central nervous system depressants may increase heat vulnerability through impaired alertness, reduced behavioral responsiveness, increased fall risk, respiratory or cognitive effects, and reduced capacity to recognize or respond to heat stress [23,57,58,59]. In addition, substance use may increase exposure to outdoor heat, impair hydration, worsen sleep, disrupt judgment, and interact with other psychiatric symptoms. These pathways are clinically important because heat protection often depends on timely behavioral changes.

From a psychiatric perspective, behavioral thermoregulation is as important as autonomic thermoregulation in maintaining homeostasis. Patients must recognize heat risk, seek a cooler environment, drink fluids, avoid exertion, adjust routines, maintain sleep, store medications appropriately, and seek help when needed. Sedation, intoxication, cognitive impairment, psychosis, depression, mania, and social isolation can disrupt this chain. Therefore, medication- and substance-related heat vulnerability should be understood as a combined biological-behavioral risk pathway.

### 10.6. Toward Climate-Informed Psychopharmacology

Climate-informed psychopharmacology does not require the abandonment of evidence-based psychiatric treatment during hot weather. Instead, it requires recognition that environmental heat modifies risk in ways that are predictable, preventable, and clinically relevant. Practical strategies include identifying patients with elevated heat vulnerability, reviewing anticholinergic and sedative burdens, monitoring hydration and renal risk, providing heatwave-specific advice, coordinating with caregivers, and integrating heat alerts into mental health services [22,23,56,57,58,59,60,61].

This framework is especially important in low- and middle-income settings, where access to air conditioning, stable housing, hydration, green spaces, transportation, and continuous healthcare may be limited. In this context, medication-related heat vulnerability may reflect not only pharmacology but also the social determinants of thermoregulatory protection. Therefore, future research should examine psychotropic medications under real-world ecological conditions, including indoor temperature, housing quality, occupational heat exposure, medication combinations, and access to clinical follow-up.

## 11. Synthesis of Biological Pathways Linking Heat Exposure and Mental Health

The biological pathways reviewed above suggest that heat exposure may influence mental health through several interacting mechanisms rather than a single causal route. Thermoregulatory strain, autonomic and cardiovascular activation, neuroinflammation, oxidative stress, mitochondrial dysfunction, blood–brain barrier disruption, hypothalamic–pituitary–adrenal axis activation, sleep and circadian disruption, neurotransmitter alterations, and psychopharmacological vulnerability may act simultaneously or sequentially, depending on exposure intensity, duration, timing, individual susceptibility, and social context.

These pathways are highly interconnected. For example, heat-related sleep disruption may amplify stress system activation and inflammatory signaling; dehydration and cardiovascular strain may worsen medication-related vulnerability; oxidative stress may reinforce neuroinflammation and endothelial dysfunction; and impaired behavioral thermoregulation may increase the risk among individuals with severe mental illness, cognitive impairment, or limited access to cooling. Thus, heat exposure should be conceptualized as a multi-system biological stressor with transdiagnostic psychiatric relevance.

To synthesize these mechanisms and clarify their clinical and research implications, Table 1 summarizes the main biological pathways linking heat exposure to mental health outcomes, their potential psychiatric relevance, key gaps in future research, and the primary type and narrative credibility of evidence supporting each pathway. Evidence credibility was classified narratively as higher, moderate, or lower according to the extent of direct human evidence, consistency across study types, and degree of reliance on experimental, preclinical, or indirect mechanistic evidence. These categories are intended as an interpretive guide rather than as a formal GRADE assessment. In this sense, the final column of Table 1 functions as a narrative level-of-evidence indicator, distinguishing pathways supported mainly by human clinical, epidemiological, or physiological evidence from pathways relying primarily on animal models, cellular studies, heatstroke or severe hyperthermia research, or indirect mechanistic evidence.

## 12. Psychiatric Outcomes Associated with Heat Exposure

### 12.1. Transdiagnostic Psychiatric Effects of Heat Exposure

Epidemiological literature suggests that heat exposure is associated with a broad range of psychiatric outcomes rather than a single diagnostic category. Systematic reviews and meta-analyses have reported positive associations between elevated ambient temperatures and mental health-related morbidity and mortality, including psychological distress, emergency department visits, psychiatric hospitalizations, substance-related outcomes, severe mental illness exacerbations, and suicide mortality [5,6,7]. This diagnostic breadth is biologically plausible because heat exposure affects multiple transdiagnostic systems, including thermoregulation, autonomic arousal, cardiovascular strain, sleep, circadian rhythms, stress biology, inflammation, oxidative stress, neurovascular integrity, and psychopharmacological vulnerability [11,12,13,14,15,16,17,18,19,20,21,22,23,31,32,33,34,35,36,37,38,39,40,41,42,43,44,45,46,47,48,49,50,51,52,53,54,55,56,57,58,59,60,61].

Large population-based studies support this transdiagnostic interpretation. Nori-Sarma et al. examined more than 3.4 million mental health-related emergency department visits among adults in the United States and found that higher warm-season temperatures were associated with increased rates of emergency department visits for any mental health condition and for several specific categories, including substance use disorders, anxiety, stress-related and somatoform disorders, mood disorders, schizophrenia-spectrum disorders, and self-harm-related encounters [62]. Mullins and White similarly reported that higher temperatures were associated with increased emergency department visits for mental illness, suicide mortality, and self-reported poor mental health in the United States [63]. In children, adolescents, and young adults, Niu et al. found that elevated ambient temperature was associated with a higher risk of mental health-related emergency department and hospital encounters across age groups from 6 to 25 years [64].

These findings suggest that heat acts less like a disorder-specific exposure and more like a multi-system stressor that reduces psychiatric resilience across diagnostic boundaries. This interpretation is consistent with the biological model proposed in the present review. Heat-related sleep loss may worsen affective regulation; autonomic activation may amplify anxiety and somatic distress; inflammatory and oxidative pathways may contribute to fatigue, low mood, and cognitive symptoms; medication-related vulnerabilities may increase clinical instability in severe mental illness; and reduced behavioral thermoregulation may increase the risk among individuals with impaired self-care, substance use, cognitive difficulties, or social isolation.

Because the epidemiological literature uses heterogeneous heat-exposure metrics and lag structures, direct quantitative comparison of effect sizes across diagnostic categories should be interpreted cautiously. Across studies, heat exposure has been operationalized using percentile-based ambient temperature, daily mean temperature, same-day mean apparent temperature, monthly average temperature, elevated temperature relative to the minimum risk temperature, and heatwave or extreme-heat definitions. Similarly, lag windows vary from same-day exposure to cumulative lag structures over several days, 7-day windows, 30-day windows, and monthly time scales. To improve comparability without imposing artificial equivalence across heterogeneous designs, Supplementary Table S2, stratifies the main heat-related psychiatric outcomes by representative study, population, exposure indicator, exposure contrast, lag or effect window, and standardized comparison notes.

### 12.2. Depression and Psychological Distress

Depression and psychological distress are among the most frequently discussed mental health outcomes related to climate change and heat exposure. Epidemiological studies have linked elevated temperatures with worse self-reported mental health, increased depressive symptoms, and mental health service use, although findings vary by setting, exposure metric, population, and outcome definition [5,6,7,8,62,63,65]. In a systematic review and meta-analysis by Thompson et al., ambient outdoor temperature was positively associated with several mental health outcomes, including worse community mental health and well-being, although the certainty of evidence varied across outcomes [7]. Similarly, Liu et al. found that hot weather was associated with poor mental health outcomes, while emphasizing heterogeneity in study design and exposure assessment [5].

Several biological pathways may explain the heat-related depressive symptoms. Sleep disruption is particularly important because elevated nighttime temperatures can reduce sleep duration and quality, and insomnia is both a symptom and a maintaining factor for depression [19,20,46,47,48,49]. Neuroinflammation and oxidative stress may also be relevant, as both inflammatory signaling and oxidative/nitrosative stress have been implicated in the pathophysiology of depression [15,34,35,37,38]. Heat-related fatigue, reduced activity, physical discomfort, dehydration, and reduced capacity for coping behaviors may further contribute to low mood and functional impairment.

However, depression-related findings should be interpreted with caution. Ambient heat may not uniformly affect all dimensions of depression. Some studies on mood symptoms suggest that temperature may have different associations with depressive and manic symptoms, and the direction of the effect may depend on seasonality, sunlight exposure, sleep patterns, and individual vulnerability. Therefore, heat-related depression should not be conceptualized as a linear phenomenon. Future studies should distinguish between acute distress, worsening depressive symptoms, sleep-mediated mood changes, and longer-term climate-related psychological burdens.

### 12.3. Anxiety, Somatic Distress, and Emotional Dysregulation

Anxiety-related outcomes are also associated with heat exposure. Nori-Sarma et al. found increased rates of emergency department visits for anxiety, stress-related, and somatoform disorders during extreme heat days [62]. Basu et al. similarly examined apparent temperature and mental health-related emergency room visits in California, contributing to evidence that heat may increase acute mental health service utilization [65]. In younger populations, Niu et al. found that adolescents with anxiety disorders showed vulnerability to elevated temperatures in stratified analyses [64].

The biological plausibility of this association is high. Heat exposure activates the autonomic and cardiovascular systems, increasing sweating, heart rate, vasodilation, and bodily discomfort [11,28,29,30]. These sensations can overlap with anxiety symptoms and may be particularly distressing for individuals with panic vulnerability, trauma-related hyperarousal, somatic symptom burden, or health-related anxiety. Heat-related sleep loss may further reduce emotional regulation and increase irritability, worry, and stress [19,20,46,47,48,49]. In addition, psychological climate stress may interact with direct thermal stress by increasing perceived threat, helplessness, and anticipatory worry [3,4,42,43,44,45].

Therefore, anxiety and somatic distress may represent a point of convergence between bodily heat strain and psychological interpretation. The same adaptive physiological response to cooling may be experienced as alarming, overwhelming, or uncontrollable in vulnerable individuals. This pathway deserves more empirical attention, particularly through studies that combine ambient temperature, indoor heat, autonomic measures, sleep data, anxiety symptoms, and health care utilization.

### 12.4. Bipolar Disorder and Mood Instability

Bipolar disorder may be particularly sensitive to heat exposure because of its close association with sleep, circadian rhythm, arousal, activity regulation, and medication safety. Elevated nighttime temperatures may impair sleep onset and continuity, whereas circadian rhythm disruption can contribute to mood instability and relapse vulnerability [19,20,46,47,48,49,50,51]. Therefore, heat-related sleep disruption may be especially relevant for manic, hypomanic, mixed, or depressive destabilization.

Epidemiological evidence specifically focused on bipolar disorder remains more limited than that for overall mental health outcomes or suicide mortality, but available studies suggest clinically relevant associations. Sung et al. reported a positive relationship between ambient temperature and bipolar disorder admissions using a national cohort of psychiatric inpatients in Taiwan, with increased relative risks associated with higher temperatures [66]. Niu et al. found vulnerability among adolescents with bipolar disorder in relation to elevated temperatures and mental health-related healthcare encounters [64]. These findings support the need for disorder-specific studies examining heat exposure, sleep disruption, circadian rhythms, and relapse risk in bipolar disorder.

Medication-related pathways are also important. Lithium safety may be affected by dehydration, renal function, sodium balance, and fluid loss during hot weather [23,57,60,61]. Antipsychotics, sedatives, antidepressants, and anticholinergic agents may further influence thermoregulation, alertness, hydration, and behavioral adaptation [22,23,56,57,58,59,60]. In this context, the clinical challenge for patients with bipolar disorder is multidimensional: heat may affect sleep and circadian stability while modifying medication-related risks and self-care demands.

### 12.5. Schizophrenia-Spectrum Disorders and Severe Mental Illness

Schizophrenia-spectrum disorders and severe mental illnesses are consistently identified as contexts of heightened heat vulnerability [21,22,23,62]. Nori-Sarma et al. found increased emergency department visits for schizophrenia, schizotypal, and delusional disorders during extreme heat days [62]. Niu et al. reported vulnerability among young adults with psychosis-related disorders during elevated temperature periods [64]. Systematic evidence also suggests that individuals with mental illness are at an increased risk of negative health effects during extreme heat events [21].

Several biological and clinical mechanisms may underlie this vulnerability. Antipsychotic medications may impair thermoregulation, increase sedation, reduce sweating through anticholinergic effects, contribute to orthostatic hypotension, and reduce behavioral responsiveness [22,23,56,57,58,59]. Severe mental illness may also be associated with reduced self-care, cognitive impairment, social isolation, homelessness, poverty, substance use, and comorbid medical illnesses, all of which can increase exposure or reduce adaptive capacity during heatwaves [21,22,23]. Heat-related sleep disruption, dehydration, and autonomic strain may further increase the risk of relapse or acute behavioral deterioration.

The concept of behavioral thermoregulation is especially important in severe mental illness. Avoiding heat exposure requires risk recognition, seeking cooler environments, drinking fluids, modifying routines, preserving sleep, storing medications appropriately, and accessing healthcare when needed. Symptoms such as disorganization, negative symptoms, cognitive impairment, paranoia, mood instability, intoxication, and sedation may impair these protective behaviors. Thus, heat vulnerability in schizophrenia-spectrum disorders is not only pharmacological but also neurocognitive, behavioral, and social.

### 12.6. Substance Use Disorders

Heat exposure may also be associated with substance-related effects. In a national study by Nori-Sarma et al., extreme-heat days were associated with increased emergency department visits for substance use disorders [62]. More recently, Jhang et al. conducted a systematic review and meta-analysis of extreme ambient temperatures and emergency healthcare service utilization due to substance use disorders, reporting an increased risk of substance use disorder-related emergency department visits during extremely high temperatures, although heterogeneity across studies was substantial [67].

Several mechanisms may contribute to this association. Heat exposure may increase physiological discomfort, sleep disruption, irritability, impulsivity, dehydration, and social stress, which may worsen the vulnerability of people with substance use disorders. Substance use may also impair thermoregulation, judgment, hydration, sleep, and behavioral adaptation. In addition, intoxication or withdrawal may reduce the ability to recognize heat risk or seek help. These pathways are particularly relevant for socially vulnerable groups, outdoor workers, unhoused individuals, and people with comorbid mental illnesses.

This area requires careful interpretation and consideration. Existing evidence often relies on healthcare utilization data rather than direct measurements of substance consumption, craving, relapse, or environmental exposure. Future studies should examine substance-specific pathways, including alcohol, stimulants, opioids, sedatives, and polysubstance use, while considering housing, occupational exposure, comorbid psychiatric disorders, and access to cooling and healthcare.

### 12.7. Suicide Mortality and Self-Harm-Related Outcomes

The association between heat exposure and suicide mortality is among the most consistently reported findings in climate-related mental health research [6,7,9,68,69]. Thompson et al. found that the strongest evidence in the earlier literature concerned increased suicide risk during periods of high ambient temperatures [6]. Burke et al. reported that increases in monthly average temperature were associated with higher suicide rates in both the United States and Mexico [9]. Heo et al. conducted a systematic review and meta-analysis of suicide risk associated with short-term exposure to ambient temperature and air pollution, supporting an association between temperature and suicide risk [68]. More recent systematic syntheses have identified high temperatures as an important climate-related factor associated with suicidal behavior and suicide mortality [69].

However, this association must be interpreted within a multifactorial framework. Suicide is not caused by temperature alone; rather, heat may contribute to population-level risk through interacting pathways, such as sleep disruption, irritability, impulsivity, psychological distress, substance use, social stress, reduced access to support, and biological stress responses [7,9,19,20,42,43,44,45,46,47,48,49,62,68,69]. Neuroinflammation, oxidative stress, HPA-axis dysregulation, and neurotransmitter changes may also contribute to vulnerability; however, direct mechanistic evidence linking these pathways to heat-related suicide outcomes remains limited [14,15,16,34,35,36,37,38,52,53,54,55].

Clinically and in public health, the key implication is prevention. Heat-health plans should recognize people with mental disorders as a potentially vulnerable group and integrate mental health into warning systems, outreach, clinical monitoring, medication review, sleep protection, and access to crisis care [3,21,22,23,70]. Future research should prioritize designs that combine high-resolution temperature exposure, nighttime heat, sleep measures, psychiatric history, medication exposure, social vulnerability, and population-level outcomes. This would help clarify whether heat-related suicide risk is primarily mediated through sleep, acute distress, substance-related crises, behavioral dysregulation, or other mechanisms.

## 13. Synthesis of Disorder-Specific Vulnerability

Overall, the psychiatric effects of heat exposure appear to be transdiagnostic but not uniform across different populations. Depression may be influenced by sleep loss, inflammation, oxidative stress, fatigue, and reduced coping capacities. Anxiety may be amplified by autonomic arousal, bodily discomfort, and perceived threats. Bipolar disorder may be particularly sensitive to sleep and circadian disruption, as well as medication-related dehydration risk. Schizophrenia-spectrum disorders may involve thermoregulatory impairment, antipsychotic exposure, cognitive and behavioral vulnerabilities, and social disadvantages. Substance use disorders may be affected by impaired judgment, dehydration, sleep disruption, behavioral dysregulation, and emergency healthcare needs. Suicidal mortality and self-harm-related outcomes likely reflect multiple interacting pathways rather than a single biological mechanism [71].

The disorder-specific evidence reviewed above suggests that heat exposure may influence psychiatric outcomes through both shared and diagnosis-specific mechanisms. Although mechanisms such as sleep disruption, autonomic arousal, inflammation, oxidative stress, and medication-related vulnerability cut across diagnostic categories, their clinical expression may differ according to the underlying disorder, treatment profile, behavioral adaptation capacity, and social context. Accordingly, Table 2 should be interpreted as a clinical and mechanistic synthesis rather than as a quantitative ranking of temperature effects across diagnoses. The exposure indicators and lag windows used in the main epidemiological studies are summarized separately in Supplementary Table S2. Table 2 summarizes the heat-sensitive mechanisms and clinical implications across major psychiatric conditions.

## 14. Contextual Modifiers and Susceptible Population Groups of Biological Heat Vulnerability

### 14.1. Vulnerability as a Biological and Clinical Context-Dependent Construct

Heat-related psychiatric vulnerability is not determined by temperature alone. The same ambient temperature may have different biological and mental health consequences depending on age, medical comorbidity, medication exposure, hydration status, sleep recovery, housing quality, occupational conditions, access to cooling, and baseline psychiatric risks. Therefore, vulnerability should be understood as a context-dependent interaction between heat exposure and individual biological susceptibility.

To make the subgroup-oriented synthesis explicit, susceptible populations were organized across six overlapping vulnerability dimensions: age and developmental stage; psychiatric and clinical status; psychotropic medication and medical comorbidity; socioeconomic and geographic vulnerability; housing, urban, and occupational heat exposure; and access to cooling, health care, and social support. This framework includes older adults; children, adolescents, and young adults; people with severe mental illness; individuals receiving heat-sensitive psychotropic medications; people with cognitive impairment or substance use disorders; low-income populations; people living in poorly cooled or overcrowded housing; outdoor workers; and populations in low- and middle-income countries. These categories are not mutually exclusive. Rather, heat-related vulnerability may increase when several dimensions overlap, such as severe mental illness, antipsychotic exposure, cardiometabolic comorbidity, poverty, social isolation, and limited access to cooling or health care.

Broader climate and mental health research support the relevance of these contextual modifiers. Climate-related mental health effects occur through direct exposure, such as heatwaves and extreme weather events, and through indirect pathways that affect daily functioning, resources, displacement, and social stability [3,4,73,74]. A multinational study of climate anxiety, coping, and psychosocial responses found that climate-related functional disruption was an important predictor of climate anxiety, supporting the idea that climate-related distress is shaped not only by exposure but also by perceived interference with daily life and adaptive capacity [73]. Similarly, climate change has been conceptualized as a threat multiplier that intensifies pre-existing inequities, conflict pathways, displacement, and mental health vulnerability [74].

In the present review, these contextual factors are primarily considered as modifiers of biological heat vulnerability. Thermoregulatory strain depends not only on external temperature but also on cardiovascular reserve, sweating capacity, hydration, renal function, acclimatization, and behavioral adaptation [11,75,76]. Sleep disruption depends not only on nighttime temperature but also on indoor heat, ventilation, crowding, noise, and access to cooling systems [19,20]. Medication-related vulnerability depends not only on pharmacology but also on dehydration risk, renal function, monitoring, continuity of care, and the ability to recognize early symptoms of heat-related illness [22,23,56,57,58,59,60,61]. Thus, contextual conditions determine whether heat-related biological stress is buffered, tolerated or amplified. The following subsections therefore organize the evidence across major susceptible population groups and contextual vulnerability domains, including age, severe mental illness, socioeconomic and geographic vulnerability, housing, occupational exposure, access to cooling, and implications for climate-informed psychiatric care, rather than treating heat exposure as a uniform risk across all individuals.

### 14.2. Older Adults

Older adults are among the most heat-vulnerable groups because aging is associated with reduced thermoregulatory reserve, altered thirst perception, reduced sweating efficiency, cardiovascular limitations, multi-morbidity, polypharmacy, and a higher risk of dehydration [75,76]. These vulnerabilities are highly relevant to mental health issues. Older adults with depression, cognitive impairment, psychosis, substance use, neurocognitive disorders, social isolation, or limited mobility may have a reduced capacity to recognize heat risk, maintain hydration, adjust routines, or seek assistance during heatwaves.

Heat-related sleep disruption may be particularly relevant in older adults. Wearable-based evidence suggests that the sleep-eroding effects of warmer nights are larger among older individuals, and systematic review evidence indicates that ambient heat can impair sleep quantity and quality [19,20]. As sleep disturbance is linked to affective symptoms, cognition, irritability, and daytime functioning, nighttime heat may act as a clinically important pathway for psychiatric deterioration in older populations.

Medication exposure adds another layer of vulnerability to this population. Older adults are more likely to receive multiple medications, including psychotropics, antihypertensives, diuretics, anticholinergics, sedatives, and other drugs that may affect hydration, sweating, blood pressure, cognition, renal function, and thermoregulation [22,23,56,57,58,59,60,61]. In psychiatric care, this makes heatwave preparedness particularly important for older patients receiving antipsychotics, lithium, sedatives, antidepressants with anticholinergic properties, and complex medication regimens. Clinical strategies should include anticipatory counseling, hydration planning, medication review, caregiver involvement, and attention to housing and cooling access for older adults.

### 14.3. Children, Adolescents, and Young Adults

Children, adolescents, and young adults may also be vulnerable to heat-related mental health effects, although the mechanisms differ from those affecting older adults. Developmental stage, sleep needs, school or university demands, social dependence, emotional regulation, outdoor activity, and family resources may shape heatwave vulnerability. A systematic review and meta-analysis focusing on children and adolescents found associations between heat exposure and mental health or suicide-related outcomes, highlighting the need to consider early life vulnerability in climate-related psychiatric research [10]. Niu et al. reported associations between elevated ambient temperatures and mental health-related emergency department and hospital encounters among children, adolescents, and young adults [64].

Sleep and circadian pathways are likely to be especially important in the younger population. Adolescents experience developmental changes in sleep timing, circadian preference, academic schedules, screen exposure, and emotional regulation. Elevated nighttime temperatures may further impair sleep onset and sleep continuity, potentially worsening mood symptoms, anxiety, irritability, daytime fatigue, and cognitive performance [19,20,46,47,48,49]. These effects may be greater in settings where housing conditions, school infrastructure, or access to cooling are limited due to financial constraints.

Heat vulnerability in young people is also shaped by familial and community contexts. Children and adolescents depend on caregivers, schools, and local systems for protection from heat events. When families face poverty, overcrowding, food insecurity, limited healthcare access, or unsafe housing, their capacity to reduce heat exposure may be constrained. Therefore, youth-focused climate mental health strategies should include schools, pediatric services, families, and community organizations rather than placing the responsibility solely on individual behavior.

### 14.4. People with Severe Mental Illness

Individuals with severe mental illness represent a priority group for heat-related psychiatric prevention. Their vulnerability reflects the convergence of biological, pharmacological, behavioral, cognitive and social factors. Severe mental illness may be associated with impaired self-care, social isolation, homelessness, poverty, cognitive dysfunction, reduced insight, substance use, medical comorbidity, and reduced access to preventive health care [21,22,23]. These factors can increase heat exposure and reduce the capacity for behavioral thermoregulation.

Psychotropic medication exposure is central to this group. Antipsychotics, anticholinergic agents, sedatives, antidepressants, mood stabilizers, and polypharmacy may influence thermoregulation, sweating, sedation, blood pressure, hydration, renal function, and behavioral responsiveness [22,23,56,57,58,59,60,61]. Therefore, heat-related risks in severe mental illness are not limited to environmental exposure; they emerge from the interaction between treatment, physiology, symptoms, and context. For example, a patient with a schizophrenia-spectrum disorder taking antipsychotic medication, living alone in poorly ventilated housing, and experiencing cognitive impairment may face a much higher risk than a patient with similar medication exposure but strong social support and reliable cooling access.

Evidence from a South American emergency psychiatric setting reinforces the importance of examining temperature-related psychiatric presentations in resource-limited and under-represented regions [72]. Such evidence is relevant because heat-related biological vulnerability may be shaped by real-world clinical conditions, including limited surveillance systems, constrained mental health services, medication access, housing quality and heat adaptation capacity. Future research should examine severe mental illness in real-world heat contexts, including housing, medication, comorbidities, service access, and social support.

### 14.5. Low- and Middle-Income Countries and Underrepresented Regions

Low- and middle-income countries may face disproportionate heat-related mental health burdens because climatic exposure often interacts with constrained infrastructure, limited cooling access, informal housing, occupational heat, urban heat islands, weaker health systems, and under-resourced mental health services [2,3,75,76,77,78]. These conditions may amplify the biological pathways. High nighttime temperatures in poorly ventilated housing may worsen sleep; outdoor work may increase dehydration and exhaustion; limited healthcare access may delay the recognition of medication-related complications; and reduced access to cooling may increase thermoregulatory strain.

The burden of heat-related health risks is also shaped by anthropogenic climate change. Vicedo-Cabrera et al. estimated that a substantial proportion of warm-season heat-related mortality across multiple countries was attributable to human-induced climate change, with burdens evident across continents [78]. Although this study focused on mortality rather than psychiatric outcomes, it underscores the broader point that current heat exposure is being intensified by climate change. For psychiatry, this means that heat-related mental health vulnerability should be understood as part of climate adaptation planning, particularly in regions where biological susceptibility and limited protective infrastructure may overlap with each other.

Geopsychiatry offers a useful framework for situating biological risks in place-based contexts. Within this framework, heat exposure is not only a meteorological variable but also a geographically distributed stressor, whose psychiatric effects depend on infrastructure, inequity, healthcare capacity, housing, medication continuity, and access to adaptive resources [73,74]. In the present review, this perspective is used to emphasize that biological heat vulnerability is socially patterned and geographically uneven rather than to shift the focus away from biological mechanisms.

### 14.6. Housing, Urban Heat, Occupational Exposure, and Access to Cooling

Housing quality is a major contextual modifier of heat-related mental health issues. Indoor heat may differ substantially from outdoor temperatures, especially in poorly insulated, overcrowded, or poorly ventilated homes. Elevated indoor nighttime temperatures may impair sleep recovery, even when daytime heat exposure is not extreme. This is particularly relevant for individuals with depression, anxiety, bipolar disorder, psychosis, neurocognitive impairment, or medication-related heat sensitivity, as sleep and recovery are central to psychiatric stability.

Urban environments may also intensify heat exposure through heat island effects, reduced vegetation, high building density, asphalt surfaces, air pollution, and limited access to green spaces. Socioeconomic disparities determine who is most exposed to these conditions. Gronlund reviewed evidence that racial and socioeconomic disparities in heat-related health effects may be mediated by access to air conditioning or cool environments, comorbidities, medication use, occupation, and urban heat island effects [77]. These mechanisms are highly relevant to mental health because they influence both biological exposure and adaptive capacity.

Occupational heat exposure is another important modifier. Outdoor, agricultural, and construction workers, street vendors, emergency responders, and people working in poorly ventilated indoor environments may experience repeated heat stress, dehydration, fatigue, and sleep disruption. Heat stress can also reduce physical work capacity and motor-cognitive performance, with implications for safety, productivity, and mental well-being [75]. Therefore, psychiatric research should include occupational exposure and socioeconomic position as core variables rather than treating heat exposure uniformly across populations.

### 14.7. Implications for Climate-Informed Psychiatric Care

The vulnerability modifiers reviewed above suggest that climate-informed psychiatric care should be proactive, stratified and context-sensitive. Patients with elevated heat vulnerability may include older adults, people with severe mental illness, individuals taking thermoregulation-impairing medications, people with bipolar disorder receiving lithium, patients with cognitive impairment, those with substance use disorders, people experiencing homelessness, outdoor workers, and individuals living in poorly cooled housing.

Clinical practice should include heat risk assessment, medication review, hydration counseling, sleep protection planning, caregiver involvement, and coordination with primary care or community services during heatwaves. Public health systems should integrate mental health into heat-health action plans, including outreach to psychiatric populations, monitoring of emergency presentations, attention to nighttime heat, and support for cooling access during heatwaves. In low-resource settings, interventions may need to prioritize feasible measures, such as hydration planning, shaded community spaces, early warning communication, medication safety education, and social support networks.

Overall, heat-related psychiatric vulnerability emerges from the interaction between biological susceptibility and contextual exposures. A person’s risk depends not only on temperature but also on thermoregulatory reserve, medication profile, sleep recovery, hydration, comorbid illness, housing, occupational exposure, social support, and whether healthcare systems can anticipate rather than merely respond to heat-related crises.

## 15. Toward an Integrative Biological Model

### 15.1. Heat Exposure as a Multi-System Biological Stressor

The evidence reviewed in the previous sections suggests that heat exposure should be conceptualized as a multi-system biological stressor rather than as a simple environmental exposure. Heat challenges thermoregulation, activates autonomic and cardiovascular responses, disrupts hydration and sleep, engages stress-response systems, and may contribute to inflammatory, oxidative, neurovascular, and neurochemical changes [11,12,13,14,15,16,17,18,19,20,21,22,23,31,32,33,34,35,36,37,38,39,40,41,42,43,44,45,46,47,48,49,50,51,52,53,54,55,56,57,58,59,60,61]. These pathways are highly interconnected and may converge on psychiatric outcomes through their effects on arousal, cognition, emotional regulation, fatigue, impulsivity, sleep stability, medication safety, and behavioral adaptation.

This model helps explain why heat exposure is associated with heterogeneous psychiatric effects. Rather than acting through a disorder-specific pathway, heat may lower the threshold for symptom exacerbation across diagnostic categories. For example, sleep disruption may worsen depression, anxiety, bipolar disorder, and psychosis; autonomic activation may amplify anxiety and somatic distress; dehydration may increase medication-related risk; and inflammatory or oxidative processes may contribute to fatigue, cognitive dysfunction, and affective symptoms. The same heat exposure may produce different psychiatric consequences depending on individual biology, diagnosis, medication, and environmental context.

Importantly, the biological effects of heat are unlikely to occur in isolation from other environmental factors. Heatwaves may simultaneously increase nighttime temperatures, reduce sleep, produce dehydration, increase cardiovascular strain, alter medication tolerability, and increase psychological stress. In vulnerable individuals, these pathways may reinforce each other. For instance, poor sleep may increase stress system activation and inflammatory signaling, dehydration may increase cardiovascular and renal strain, medication-related sedation may reduce behavioral thermoregulation, and cognitive impairment may reduce the likelihood of protective actions. This supports an integrated model in which heat-related psychiatric vulnerability emerges from the interaction of biological and contextual pathways.

### 15.2. Acute and Chronic Pathways

The temporal dimension of heat exposure is central to the proposed model. Acute heat exposure, such as heatwaves or sudden temperature spikes, may precipitate short-term psychiatric deterioration via rapid physiological strain. Potential acute pathways include autonomic arousal, dehydration, sleep loss, medication intolerance, agitation, anxiety, delirium vulnerability, substance-related crises, and emergency mental health presentations [5,6,7,11,21,22,23,56,57,58,59,60,61].

Chronic or repeated heat exposure may operate through different but overlapping mechanisms. Persistent high temperatures, elevated nighttime temperatures, repeated occupational heat exposure, and insufficient recovery may contribute to the cumulative biological burden. Over time, repeated thermoregulatory strain may interact with allostatic load, chronic sleep disruption, inflammatory priming, oxidative stress, reduced resilience, and worsening of comorbid medical conditions [19,20,36,37,38,39,40,41,42,43,44,45]. These chronic pathways are particularly relevant in regions where heat exposure is seasonal, prolonged, or intensified by climate change, urban heat islands, housing quality, and limited access to cooling.

The distinction between acute and chronic pathways has important implications for research. Acute pathways may be best captured through time-series, case-crossover, emergency department, and hospitalization studies. Chronic pathways require longitudinal designs that incorporate repeated psychiatric assessments, indoor and outdoor temperature exposure, nighttime heat, sleep metrics, biomarkers, medication data and contextual modifiers. Without this distinction, studies may overlook important differences between short-term destabilization and cumulative biological vulnerability.

### 15.3. Biological Vulnerability and Contextual Amplification

The proposed model also emphasizes that biological vulnerability is contextually amplified. Heat exposure does not affect all individuals equally. Older adults, children and adolescents, people with severe mental illness, individuals taking psychotropic medications, people with medical comorbidities, outdoor workers, and those living in poorly cooled or overcrowded housing may experience greater biological strain under similar thermal conditions [10,19,20,21,22,23,56,57,58,59,60,61,64,72,73,74,75,76,77,78].

This does not reduce the biological relevance of the model. Rather, it clarifies that contextual conditions determine the intensity, duration, and recoverability of the biological heat stress. Access to cooling may protect sleep and reduce the cardiovascular strain. Access to hydration may reduce renal and medication-related risks. Housing quality may determine nighttime heat exposure. Social support may facilitate behavioral thermoregulation. Healthcare access may determine whether early signs of medication toxicity, dehydration, delirium, and psychiatric relapse are recognized and treated. In this sense, social and environmental conditions modify biological pathways.

This is where geopsychiatry becomes relevant. Place, climate, infrastructure, social inequity, and healthcare capacity shape the biological experience of heat exposure [73,74]. The same mechanism, such as sleep disruption or dehydration, may have different consequences depending on whether the person has access to air conditioning, stable housing, safe water, medication monitoring, family support, or emergency care. Thus, biologically grounded climate psychiatry requires attention to both mechanisms and context.

### 15.4. Proposed Integrative Model

Based on the evidence reviewed, heat exposure may be understood as acting through three interacting levels: environmental exposure, biological response, and psychiatric expression. The first level is environmental exposure, which includes ambient heat, heatwaves, nighttime heat, humidity, indoor heat, urban heat islands, and occupational heat. These exposures are not interchangeable. Daytime maximum temperature may capture acute thermal stress, whereas nighttime heat may be particularly relevant to sleep loss and impaired physiological recovery; indoor heat may better reflect household exposure; and occupational heat may capture repeated physical and thermal strain in working populations [1,2,3,4,5,6,7,8,9,10,11,19,20,75,76,77,78]. Therefore, the model begins with the recognition that heat exposure is multidimensional and that biologically relevant exposure may differ from outdoor ambient temperature alone.

The second level is the biological response, including thermoregulatory strain, autonomic activation, dehydration, neuroinflammation, oxidative stress, mitochondrial dysfunction, blood–brain barrier changes, HPA-axis activation, sleep and circadian disruption, neurotransmitter alterations, and psychopharmacological vulnerability [11,12,13,14,15,16,17,18,19,20,21,22,23,26,27,28,29,30,31,32,33,34,35,36,37,38,39,40,41,42,43,44,45,46,47,48,49,50,51,52,53,54,55,56,57,58,59,60,61]. These mechanisms should not be interpreted as isolated or mutually exclusive pathways. Rather, they may reinforce one another. For example, dehydration may increase cardiovascular and renal strain; sleep disruption may amplify stress and inflammatory responses; oxidative stress may interact with neuroinflammation and endothelial dysfunction; and psychotropic medication exposure may alter thermoregulation, hydration, sedation, renal safety, and behavioral adaptation [14,15,16,17,18,19,20,21,22,23,31,32,33,34,35,36,37,38,39,40,41,42,43,44,45,46,47,48,49,50,51,52,53,54,55,56,57,58,59,60,61].

The third level is psychiatric expression, including psychological distress, depression, anxiety, mood instability, psychotic vulnerability, substance-related crises, neurocognitive symptoms, delirium vulnerability, and suicide-related outcomes [5,6,7,8,9,10,21,62,63,64,65,66,67,68,69,70,71,72]. These outcomes are transdiagnostic but not uniform. The same environmental exposure may contribute to different psychiatric outcomes depending on the dominant biological pathway, baseline diagnosis, medication exposure, developmental stage, comorbid illness, and social context. For example, sleep and circadian disruption may be especially relevant to mood instability, autonomic arousal may amplify anxiety and somatic distress, dehydration and renal vulnerability may be particularly relevant for lithium-treated patients, and impaired behavioral thermoregulation may increase vulnerability among people with severe mental illness or cognitive impairment [19,20,21,22,23,50,51,56,57,58,59,60,61,64,66,72].

These levels are modified by age, comorbidities, medication exposure, severe mental illness, housing quality, occupational exposure, access to cooling, healthcare capacity, and social support [21,22,23,56,57,58,59,60,61,73,74,75,76,77,78]. These modifiers do not simply add external context to the model; they shape the biological experience of heat exposure itself. Housing quality influences indoor and nighttime heat; occupational conditions influence repeated thermal strain; medication exposure modifies thermoregulation, hydration, renal function, and sedation; and social support or healthcare access determines whether early signs of heat-related deterioration are recognized and addressed [21,22,23,56,57,58,59,60,61,73,74,75,76,77,78]. Thus, heat-related psychiatric risk emerges from the interaction between environmental exposure, biological susceptibility, clinical status, and adaptive resources.

Therefore, the model avoids a simple linear pathway in which heat exposure directly produces psychiatric outcomes. Instead, it proposes a dynamic system in which environmental heat acts on multiple biological systems, these systems interact with one another, and their psychiatric consequences are shaped by vulnerability modifiers and protective resources. This integrative model is consistent with the broader climate mental health literature, which emphasizes that climate-related mental health outcomes arise from the interaction of direct environmental exposures, biological responses, social conditions, and healthcare capacity [1,2,3,4,73,74]. It also provides a structure for organizing future research: exposure studies can refine the first level, biomarker and physiological studies can test the second level, psychiatric epidemiology and clinical studies can clarify the third level, and geopsychiatric and public health research can examine the contextual modifiers that determine who is most vulnerable and why.

**Figure 1:**
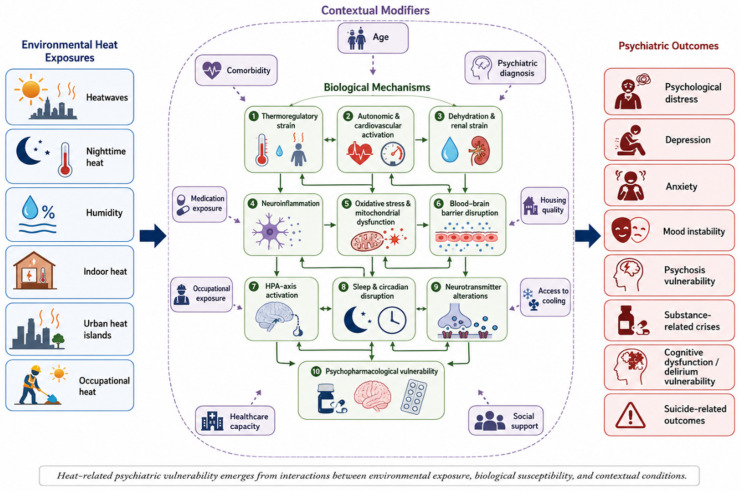
Integrative biological model of heat exposure and psychiatric outcomes. Climate-related heat exposure may affect mental health through the interaction of biological pathways, including thermoregulatory strain, autonomic and cardiovascular activation, dehydration, neuroinflammation, oxidative stress, mitochondrial dysfunction, blood–brain barrier disruption, HPA-axis activation, sleep and circadian disruption, neurotransmitter alterations, and psychopharmacological vulnerability. Large blue arrows indicate the overall progression from environmental heat exposures to biological mechanisms and psychiatric outcomes. Solid green arrows indicate proposed directional interactions among biological mechanisms, whereas double-headed green arrows indicate reciprocal or bidirectional interactions. Dashed purple arrows indicate the modifying influence of contextual factors on these biological pathways. These pathways are modified by age, comorbidities, psychiatric diagnoses, medication exposure, housing, occupational exposure, access to cooling, healthcare capacity, and social support. Psychiatric outcomes may include psychological distress, depression, anxiety, mood instability, psychosis, substance-related crises, cognitive dysfunction, delirium, and suicide-related outcomes [1,2,3,4,5,6,7,8,9,10,11,12,13,14,15,16,17,18,19,20,21,22,23,26,27,28,29,30,31,32,33,34,35,36,37,38,39,40,41,42,43,44,45,46,47,48,49,50,51,52,53,54,55,56,57,58,59,60,61,62,63,64,65,66,67,68,69,70,71,72,73,74,75,76,77,78].

Figure 1 presents this integrative model of heat exposure, biological mechanisms, contextual modifiers, and psychiatric outcomes.

## 16. Research Gaps and Future Directions

### 16.1. Moving from Epidemiological Association to Mechanistic Evidence

Research on heat exposure and mental health has expanded rapidly; however, much of the existing literature remains epidemiological. Studies have linked high ambient temperatures, heatwaves, and extreme heat with mental health-related emergency visits, psychiatric hospitalizations, psychological distress, substance-related outcomes, and suicide mortality [5,6,7,8,9,10,62,63,64,65,66,67,68,69,70,71,72]. However, few studies have directly measured the biological pathways that may explain these associations. Consequently, the field is currently stronger in documenting associations than in identifying causal mechanisms.

Future research should focus on integrated mechanistic designs. Such studies should combine environmental exposure data, psychiatric assessments, and biological measures. Relevant exposure variables include daily maximum temperature, nighttime minimum temperature, apparent temperature, humidity, indoor temperature, occupational exposure, urban heat island intensity, and duration of heat exposure. Relevant biological variables include inflammatory markers, oxidative stress markers, cortisol, autonomic indices, hydration status, renal function, sleep and circadian measures, and medication exposure [11,12,13,14,15,16,17,18,19,20,21,22,23,31,32,33,34,35,36,37,38,39,40,41,42,43,44,45,46,47,48,49,50,51,52,53,54,55,56,57,58,59,60,61]. Without this integration, heat-related psychiatric outcomes remain difficult to distinguish from broader climate-related distress, socioeconomic stress, or seasonal variation.

Current evidence from heatstroke, severe hyperthermia, cellular models, and experimental physiology supports mechanistic plausibility [17,31,32,33,34,35,36,37,38,39,40,41], but psychiatric research now requires studies in real-world populations exposed to ordinary heatwaves, repeated nighttime heat, indoor heat, and chronic occupational heat. This is especially important for people with severe mental illness, bipolar disorder, older adults, people taking psychotropic medications, and individuals living in low-resource settings.

### 16.2. Improving Exposure Assessment

A key limitation in this field is the measurement of exposure. Many studies use outdoor ambient temperature data from meteorological stations, which are often aggregated at the city, regional, or daily levels. Although useful for population-level analyses, this approach may not capture the biologically relevant exposure experienced by individuals. Indoor temperature, nighttime heat, humidity, radiant heat, air movement, clothing, activity level, housing quality, and access to cooling can all modify physiological heat load [11,19,20,75,76,77,78].

Future studies should improve exposure assessment by incorporating high-resolution meteorological data, indoor temperature monitoring, wearable sensors, geospatial exposure modeling, and individual-level data on housing, occupation, mobility, and cooling access. Nighttime temperature deserves special attention because it may reduce physiological recovery and impair sleep, a central mediator of psychiatric vulnerability [19,20,46,47,48,49,50,51]. Similarly, occupational heat exposure should be measured directly whenever possible rather than inferred from job category alone.

Methodologically, distributed lag non-linear models remain highly relevant because the effects of heat may be non-linear and delayed across several days [79]. Future psychiatric studies should use designs capable of distinguishing between immediate effects and lagged and cumulative exposure. This is particularly important for psychiatric emergencies, hospitalizations, sleep disruption, medication-related complications, and suicide-related outcomes.

### 16.3. Biomarkers and Biological Signatures

The next generation of heat–mental health research should identify the biological signatures of heat-related psychiatric vulnerability. Candidate biomarkers include inflammatory markers such as interleukin-6, C-reactive protein, tumor necrosis factor-α, and interleukin-1β; oxidative stress and nitrosative stress markers; mitochondrial function indicators; cortisol and other HPA-axis measures; autonomic indices such as heart rate variability; hydration and renal markers; and neurovascular or blood–brain barrier-related indicators [14,15,16,17,18,31,32,33,34,35,36,37,38,39,40,41,42,43,44,45].

These biomarkers should not be studied in isolation from one another. A multi-marker approach is more consistent with the biological model proposed in this review. For example, heat-related psychiatric vulnerability may simultaneously involve sleep disruption, cortisol dysregulation, inflammatory activation, oxidative stress, and medication-related dehydration. Studies that measure only one pathway may underestimate the multi-system nature of heat stress. Longitudinal repeated-measures designs are especially valuable because they can track within-person changes before, during, and after heatwaves.

Neuroimaging and digital phenotyping may also contribute to future studies. Neuroimaging can help examine whether repeated heat exposure is associated with neurovascular, inflammatory, or structural brain changes in vulnerable populations. Wearable devices can capture sleep, activity, heart rate, and physiological recovery during heat exposure. Smartphone-based ecological momentary assessment can measure mood, anxiety, irritability, fatigue, perceived heat stress, hydration behavior, and coping responses in real time. Together, these tools can help connect environmental exposure with biological responses and psychiatric experiences.

### 16.4. Psychopharmacology and Medication Safety

Medication-related heat vulnerability remains a significant research gap. Although psychotropic medications are frequently mentioned in heat-health guidance, direct evidence varies substantially by medication class, dose, patient population, and exposure conditions [22,23,56,57,58,59,60,61]. Future studies should move beyond broad medication categories and examine specific mechanisms, including anticholinergic burden, sedation, sweating, thirst, cardiovascular response, renal function, lithium levels, electrolyte balance, and cognitive capacity to respond to heat.

Lithium deserves particular attention because heat exposure, sweating, dehydration, renal function, and sodium balance may influence its serum concentrations and toxicity risk [60,61]. Antipsychotics also require focused study because they may affect thermoregulation, sedation, dopamine signaling, anticholinergic burden, orthostatic tolerance, and behavioral adaptation [22,23,56,57,58,59]. Antidepressants, benzodiazepines, hypnotics, and medications used for comorbid medical illnesses should also be evaluated under real-world heat conditions.

Clinically useful research should identify which patients require additional monitoring during heatwaves, which medication combinations are most concerning, and which preventive strategies are feasible. These strategies may include heat-risk counseling, hydration planning, caregiver involvement, temporary monitoring of renal function or lithium levels in selected cases, review of anticholinergic burden, and integration of heat alerts into psychiatric follow-up.

### 16.5. Severe Mental Illness and High-Risk Clinical Populations

Individuals with severe mental illness should be prioritized in future heat-related psychiatric research. Their vulnerability is biologically and socially complex, involving medication exposure, impaired behavioral thermoregulation, cognitive dysfunction, comorbid medical illnesses, reduced access to care, homelessness, and social isolation [21,22,23,56,57,58,59,60,61,72]. However, studies often broadly group psychiatric diagnoses or rely on administrative categories that may not capture the mechanisms.

Future studies should examine severe mental illness through detailed clinical phenotyping. Key variables include diagnosis, symptom severity, cognitive status, functional impairment, medication regimen, anticholinergic burden, substance use, comorbid physical illness, housing conditions, social support, and access to cooling. These should be linked to heat exposure metrics and biological measures. Such designs would help identify why some patients remain stable during heatwaves, whereas others experience relapse, emergency visits, delirium vulnerability, or medication-related complications.

Bipolar disorder also deserves specific attention because of its association with sleep, circadian rhythm, lithium safety, and mood instability [50,51,60,61,66,72]. Future research should examine whether nighttime heat, reduced sleep duration, delayed sleep onset, and circadian instability mediate heat-related bipolar relapses or emergency presentations. This would provide a strong mechanistic link between environmental exposure and clinical psychiatry.

### 16.6. Interventions, Heat-Health Systems, and Clinical Translation

The ultimate goal of this research area is to understand the mechanisms and prevent avoidable psychiatric harm during heat exposure. Future intervention research should test whether mental health outcomes improve when heat-health systems include individuals in psychiatric risk groups. The World Health Organization has emphasized the need for climate-resilient health systems capable of protecting health in changing climates [80]. Heat-health warning systems and heat-health action plans provide established public health frameworks for anticipating and responding to extreme heat [81,82]. However, mental health is not always explicitly integrated into these systems of care.

Clinical translation should include psychiatric heat-risk assessment, medication review, hydration counseling, sleep-protection planning, caregiver outreach, and attention to high-risk groups. Health systems can integrate heat alerts into outpatient mental health services, community mental health teams, emergency departments, pharmacies, and primary care. For example, heatwave alerts could trigger reminders to review lithium safety, check on older adults with severe mental illness, provide sleep protection advice, and coordinate outreach for patients experiencing homelessness or social isolation.

Intervention studies should be rigorously designed and evaluated. Complex intervention frameworks can guide the development and testing of multi-component heat adaptation interventions in psychiatric care [83]. Prediction model guidance may also be useful for developing transparent heat-risk stratification tools that incorporate age, diagnosis, medication profiles, comorbidities, housing, and prior emergency service use [84]. These tools should be validated in different climates and healthcare systems before their implementation.

### 16.7. Priorities for Low- and Middle-Income Settings

Low- and middle-income settings require specific research attention because heat exposure may interact with limited cooling access, informal housing, outdoor work, under-resourced mental health systems, and weaker surveillance infrastructure [2,3,72,73,74,75,76,77,78]. Research in these settings should not simply replicate the models of high-income countries. Instead, locally feasible exposure measures, biomarkers, clinical protocols, and public health responses should be developed.

Priority areas include psychiatric emergency surveillance during heatwaves, medication safety protocols adapted to resource-limited services, sleep-focused interventions that do not depend on air conditioning, community-based hydration and cooling strategies, and the integration of mental health into disaster preparedness and climate adaptation. Studies should also examine the cultural differences in heat perception, help-seeking, family support, and coping strategies.

Equity should be the central focus. The populations most exposed to heat may also be the least represented in research. This includes people living in informal settlements, rural and agricultural workers, Indigenous communities, migrants, older adults living alone, people with severe mental illness, and those with limited access to digital tools. Therefore, future studies should prioritize inclusive recruitment and community partnerships.

### 16.8. Major Limitations of the Current Literature

The current literature on heat exposure and mental health has several important limitations that should be considered when interpreting the evidence. First, much of the available evidence is observational, including time-series, case-crossover, ecological, administrative, and population-based studies. These designs are essential for detecting population-level associations between temperature and psychiatric outcomes, but they cannot fully establish individual-level causality or identify the biological mechanisms responsible for the observed associations [5,6,7,8,9,10,62,63,64,65,66,67,68,69,70,71,72].

Second, heat exposure definitions vary substantially across studies. Some studies use daily maximum temperature, others use daily mean temperature, minimum nighttime temperature, apparent temperature, temperature percentiles, heatwave definitions, monthly averages, or deviation from the minimum risk temperature. Lag structures also differ, ranging from same-day exposure to distributed lag windows over several days or longer time scales [5,6,7,8,9,10,62,63,64,65,66,67,68,69,70,71,72,79]. This heterogeneity limits direct comparison across studies, diagnostic categories, regions, and exposure thresholds. It also makes it difficult to determine whether differences in psychiatric outcomes reflect true clinical vulnerability or methodological differences in exposure assessment.

Third, longitudinal human mechanistic studies remain limited. Most studies linking heat exposure to psychiatric outcomes do not simultaneously measure biological mediators such as sleep, hydration, renal function, autonomic activity, inflammatory markers, oxidative stress, cortisol, neurovascular indicators, or medication concentrations. As a result, many of the mechanisms discussed in this review remain biologically plausible but incompletely tested in real-world psychiatric populations. Evidence from heatstroke, experimental physiology, animal models, and cellular studies is valuable for mechanistic interpretation, but it should not be treated as direct proof that ordinary ambient heat exposure produces the same biological effects in patients with mental disorders.

Fourth, causal inference is challenging because heat exposure is embedded within complex environmental and social contexts. Heat may co-occur with air pollution, humidity, drought, wildfire smoke, occupational stress, sleep disruption, electricity insecurity, poor housing, social isolation, and reduced healthcare access. These factors may confound, mediate, or modify the association between heat and mental health. Moreover, psychiatric outcomes are influenced by baseline diagnosis, symptom severity, medication use, comorbid physical illness, substance use, socioeconomic position, and access to care. Therefore, future studies should combine stronger causal designs, improved exposure assessment, repeated individual-level measurements, biological markers, medication data, and contextual variables to clarify when, how, and for whom heat exposure contributes to psychiatric vulnerability.

## 17. Research Priorities and Translational Directions

The research gaps reviewed above indicate that the field must move from broad epidemiological associations toward mechanistic, longitudinal, and clinically actionable evidence. Future studies should integrate environmental exposure assessment, biological measurements, psychiatric outcomes, medication data, and contextual modifiers within the same design. This is particularly important for understanding whether heat-related psychiatric vulnerability is mediated by sleep disruption, inflammatory signaling, oxidative stress, HPA-axis activation, dehydration, medication-related risk, or impaired behavioral thermoregulation.

A coordinated research agenda is needed to advance heat-related psychiatry from descriptive associations to prevention and intervention. Table 3 summarizes the priority research directions in the field, including improved exposure assessment, biomarker integration, sleep and circadian monitoring, medication safety, severe mental illness, risk prediction, low- and middle-income settings, intervention development, and policy integration [14,15,16,17,18,19,20,21,22,23,31,32,33,34,35,36,37,38,39,40,41,42,43,44,45,46,47,48,49,50,51,52,53,54,55,56,57,58,59,60,61,75,76,77,78,79,80,81,82,83,84].

More specifically, future studies should prioritize prospective psychiatric cohorts followed across warm seasons, heatwaves, and recovery periods. These cohorts should include repeated assessments of psychiatric symptoms, sleep, medication exposure, hydration, renal function, substance use, housing conditions, occupational heat exposure, and access to cooling [21,22,23,56,57,58,59,60,61,75,76,77,78,79]. Wearable devices could provide objective measurements of sleep duration, sleep timing, activity, heart rate, physiological recovery, and personal thermal exposure, while indoor sensors could capture nighttime and household heat conditions more accurately than outdoor meteorological data alone [19,20,46,47,48,49,50,51,75,76,77,78,79].

Multimodal biomarker assessment should include inflammatory markers, oxidative stress indicators, cortisol or other HPA-axis measures, autonomic indices, renal and hydration markers, and medication-specific measures such as lithium concentrations when clinically appropriate [14,15,16,17,18,31,32,33,34,35,36,37,38,39,40,41,42,43,44,45,60,61]. Neuroimaging studies may help clarify whether recurrent heat exposure is associated with neurovascular, inflammatory, or structural brain changes in high-risk groups, particularly older adults and people with severe mental illness [18,40,41]. Finally, intervention studies should test feasible heat mitigation strategies in psychiatric populations, including heat-risk counseling, medication safety review, sleep-protection interventions, hydration planning, caregiver or community outreach, access to cooling, and service-level heatwave protocols [22,23,56,57,58,59,60,61,80,81,82,83,84]. These interventions should be evaluated not only for physiological outcomes but also for psychiatric symptoms, emergency visits, relapse prevention, medication safety, and acceptability in both high-income and low- and middle-income settings.

## 18. Clinical and Public Health Translation

### 18.1. From Biological Mechanisms to Clinical Risk Recognition

The biological mechanisms reviewed in this article have direct implications for psychiatric practices. Heat-related psychiatric vulnerability may arise from thermoregulatory strain, dehydration, cardiovascular activation, sleep disruption, neuroinflammatory and oxidative pathways, HPA-axis activation, medication-related risk, and impaired behavioral thermoregulation [11,19,20,21,22,23,31,32,33,34,35,36,37,38,39,40,41,42,43,44,45,46,47,48,49,50,51,52,53,54,55,56,57,58,59,60,61]. Clinicians should consider heat exposure not only as an environmental background condition but also as a potential modifier of psychiatric stability, medication safety, sleep, cognition, and help-seeking behavior.

Clinical risk recognition should begin with the identification of patients who may be more vulnerable during periods of high temperatures. These include older adults, people with severe mental illness, individuals with bipolar disorder treated with lithium, patients taking antipsychotics or medications with anticholinergic or sedative effects, people with neurocognitive disorders, individuals with substance use disorders, patients with comorbid cardiovascular or renal disease, people experiencing homelessness, and those living or working in poorly cooled environments [21,22,23,56,57,58,59,60,61,75,76]. Such risk assessments do not require complex technology. In many settings, it can begin with simple clinical questions about housing, indoor heat, sleep, hydration, access to cooling, medication adherence and social support.

Importantly, psychiatric heat risk assessment should be anticipatory. Waiting for heat-related crises to occur may result in missed opportunities for prevention. Before and during heatwaves, clinicians can review medication profiles, identify patients at risk of dehydration or medication toxicity, discuss hydration and sleep protection, and coordinate support with caregivers, community workers, primary care providers, or emergency services. These strategies are particularly relevant for patients with severe mental illness, who may have a reduced capacity for behavioral thermoregulation and may face social or cognitive barriers to self-protection [21,22,23].

### 18.2. Medication Review and Heat-Sensitive Prescribing

Medication review is one of the most actionable components of climate-informed psychiatric care. Psychotropic medications may influence heat vulnerability through their effects on sweating, thirst, sedation, cognition, autonomic regulation, cardiovascular response, renal function, and thermoregulation [22,23,56,57,58,59,60,61]. This does not imply that evidence-based psychotropic treatments should be discontinued during hot weather. Clinicians should recognize that high temperatures may alter the risk of using these medications.

Lithium requires particular attention because dehydration, sodium balance, renal function, and fluid loss may influence its serum concentrations and toxicity risk [60,61]. During high-temperature periods, selected patients may benefit from hydration counseling, education on early warning signs of toxicity, avoidance of unnecessary dehydration, and clinical monitoring when the risk is elevated. Antipsychotics also deserve specific attention because of their potential effects on thermoregulation, sedation, anticholinergic burden, cardiovascular function, and behavioral responsiveness [22,23,56,57,58,59]. Antidepressants, benzodiazepines, hypnotics, and medications for comorbid medical conditions may also modify the risk depending on patient characteristics and medication combinations.

A practical approach is to review the heat-sensitive medication burden before the hottest periods of the year. This may include identifying anticholinergic load, sedative burden, lithium use, renal risk, polypharmacy, and medications that may impair blood pressure regulation or hydration. Medication decisions should be individualized. The goal is not to reduce treatment quality but to increase safety through monitoring, counseling, and coordination of care.

### 18.3. Sleep Protection as a Heat-Adaptation Strategy

Sleep protection should be considered a core component of heat-related psychiatric prevention. Elevated nighttime temperatures can impair sleep onset, duration, and quality, and sleep disruption is strongly linked to psychiatric symptoms and relapse vulnerability [19,20,46,47,48,49,50,51]. Therefore, nighttime heat may be a clinically relevant exposure metric for heat-related psychiatric prevention.

Clinical advice should include practical strategies to reduce the nighttime heat burden, when feasible. These may include improving ventilation, reducing heat accumulation during the day, adjusting sleep routines, using cooling methods safely, maintaining hydration, reducing evening alcohol or substance use, and identifying patients whose psychiatric symptoms worsen when their sleep is disrupted. For individuals with bipolar disorder, psychosis, depression, anxiety, or substance use disorders, preserving sleep during hot periods may be especially important.

At the public health level, heat-health alerts should include information about nighttime heat and sleep. Many public messages focus on daytime heat exposure, physical exertion, and hydration. These remain essential, but psychiatric prevention also requires attention to sleep continuity, circadian rhythms, and recovery. Integrating sleep-protection guidance into heat-health messaging may be a feasible and low-cost strategy with potential psychological benefits.

### 18.4. Integrating Mental Health into Heat-Health Action Plans

Heat-health warning systems and heat-health action plans are established public health tools for reducing heat-related morbidity and mortality [81,82]. However, mental health is often underrepresented in these strategies. A climate-informed psychiatric approach explicitly includes mental health populations in heat preparedness, surveillance, outreach, and response.

Mental health integration can occur at several levels. First, heat-health systems can identify psychiatric populations at elevated risk, including people with severe mental illness, older adults with mental disorders, individuals taking heat-sensitive psychotropic medications, people with substance use disorders, and those experiencing homelessness or social isolation [21,22,23,56,57,58,59,60,61]. Second, emergency departments and psychiatric services can monitor heat-related changes in presentations, including anxiety, agitation, insomnia, mood instability, psychotic relapse, substance-related crises, delirium vulnerability, and suicide-related outcomes [5,6,7,8,9,10,62,63,64,65,66,67,68,69,70,71,72]. Third, community mental health teams can use heat alerts to trigger proactive contact with high-risk patients during heat waves.

The World Health Organization has emphasized the importance of climate-resilient health systems [80]. Psychiatric services should be included in this agenda. Climate-resilient mental healthcare requires continuity of care during extreme heat, medication safety planning, coordination with primary care, emergency preparedness, and attention to vulnerable populations during extreme heat. In resource-limited settings, feasible interventions may include simple heat-risk screening, patient education, family involvement, hydration and cooling advice, and community-based outreach programs.

### 18.5. Physiological Adaptation, Resilience, and Heat-Mitigation Strategies

A balanced climate-informed psychiatric framework should recognize that heat responses are not uniformly harmful. Physiological adaptation and acclimatization may occur through improved thermoregulatory efficiency, earlier sweating responses, cardiovascular adjustment, plasma volume adaptation, cellular repair mechanisms, and heat shock protein responses [11,32,36,75,76]. These adaptive processes may help some individuals tolerate transient or repeated heat exposure more effectively, particularly when exposure is gradual, moderate, and followed by adequate recovery. However, acclimatization is not equally available to all populations and may be limited by age, cardiovascular or renal disease, severe mental illness, psychotropic medication exposure, dehydration, sleep disruption, poor housing, occupational heat exposure, and limited access to cooling [21,22,23,56,57,58,59,60,61,75,76,77,78].

Resilience factors may reduce the psychiatric impact of heat exposure by supporting both physiological recovery and behavioral adaptation. These include reliable access to drinking water, cooling, shaded spaces, safe housing, social support, caregiver involvement, continuity of psychiatric care, medication monitoring, sleep protection, and timely access to primary care or emergency services [73,74,75,76,77,78,80,81,82]. At the public health level, heat-health warning systems, heat-health action plans, community outreach, cooling centers or cool public spaces, occupational heat protections, and climate-resilient health services can reduce exposure, improve early risk recognition, and support vulnerable groups during periods of extreme heat [80,81,82]. For psychiatric populations, these interventions are most relevant when they are linked to proactive identification of high-risk patients, medication safety planning, sleep and hydration guidance, and continuity of care during heatwaves. Thus, adaptation and resilience should be understood not only as individual biological capacities but also as outcomes shaped by social infrastructure, healthcare preparedness, and equitable access to protective resources.

### 18.6. Practical Clinical and Public Health Actions

The biological framework proposed in this review can be translated into practical applications. At the clinical level, psychiatric teams can identify heat-vulnerable patients, review psychotropic medications, provide heat-specific counseling, monitor sleep and hydration, and coordinate support during heatwave events. At the service level, clinics can create heatwave protocols for patients with severe mental illness, bipolar disorder treated with lithium, neurocognitive disorders, substance use disorders, and high medication burden. At the public health level, mental health indicators can be incorporated into heat-health warning systems and climate adaptation plans [80,81,82,85,86].

These recommendations are based on three complementary sources of support: existing heat-health and climate-resilient health-system guidance [80,81,82,85,86], empirical evidence linking heat exposure with psychiatric outcomes, vulnerable-group risk, sleep disruption, and medication-related heat vulnerability [5,6,7,19,20,21,22,23,56,57,58,59,60,61,62,63,64,65,66,67,68,69,70,71,72,75,76,87,88,89], and expert clinical and public health synthesis where direct psychiatric intervention trials remain limited [83,88,90]. Therefore, anticipatory heat planning, medication review, hydration monitoring, sleep protection, and caregiver coordination should be interpreted as precautionary and clinically grounded strategies rather than as recommendations derived from psychiatric heat-intervention trials alone.

These actions should be proportionate and feasible to be effective. Not every patient requires laboratory monitoring, medication changes, or intensive follow-up in hot weather. However, many patients may benefit from simple preventive messages: maintain hydration, protect sleep, avoid excessive heat exposure, recognize medication-related warning signs, seek help early, and ensure that someone checks on them during heatwaves. More structured plans may be warranted for high-risk patients.

Climate-informed psychiatric care should avoid placing responsibility solely on individuals. Heat vulnerability is shaped by housing, poverty, infrastructure, occupational conditions, cooling access, social support, and healthcare capacity [73,74,75,76,77,78]. Thus, public health responses should combine individual advice with structural adaptations, including cooling centers, shaded public spaces, housing improvements, occupational protections, and accessible healthcare [75,76,77,78,80,81,82,85,86,87,88,90].

### 18.7. Proposed Translational Pathway

**Figure 2:**
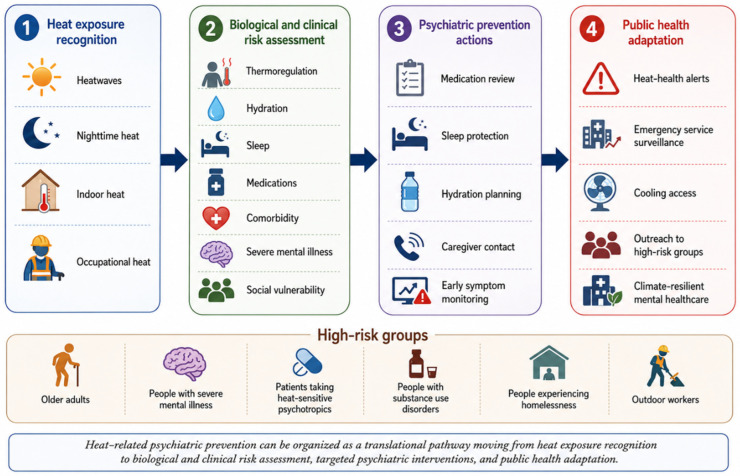
Clinical and public health translation of the heat–brain–mental health model. Heat-related psychiatric prevention can be organized as a translational pathway moving from heat exposure recognition to biological and clinical risk assessment, targeted psychiatric interventions, and public health adaptation. Key clinical actions include medication review, hydration counseling, sleep protection, caregiver outreach, monitoring of high-risk patients, and integration of mental health into heat-health warning systems. Public health actions include surveillance, community outreach, cooling access, occupational protection, and climate-resilient mental health services for vulnerable populations [80,81,82,85,86,90,91,92,93,94,95,96,97,98,99,100,101,102,103].

A translational approach should connect biological understanding with psychiatric prevention. Heat exposure should trigger the assessment of biological and clinical risks, which should guide targeted clinical actions that should be embedded within broader public health systems. Figure 2 presents the proposed pathway for translating the heat–brain–mental health model into clinical and public health practices.

The proposed pathway is based on the premise that heat-related psychiatric prevention requires movement across four linked levels: exposure recognition, biological and clinical risk assessment, targeted psychiatric action, and public health adaptation. This structure is consistent with the broader heat-health literature, which emphasizes warning systems and risk communication [80,81,82,85,86,91,92], planned heat adaptation and effective health protection [93,94], cooling strategies [95], occupational protection [96], indoor overheating prevention [97], heatwave early warning and adaptation advice [98], and the organization of heat-health warning systems across settings [99].

First, exposure recognition should include more than outdoor maximum temperature. Heat-health systems increasingly emphasize the importance of heatwaves, apparent temperature, humidity, nighttime heat, indoor overheating, urban heat islands, and occupational heat exposure [80,81,82,91,92,94,96,97,98,99]. This is particularly relevant for psychiatry because psychiatric deterioration may be driven not only by daytime thermal stress but also by nighttime heat, poor sleep recovery, indoor heat accumulation, dehydration, and repeated occupational or household heat exposure [19,20,75,76,77,78,96,97]. Therefore, psychiatric services should interpret heat alerts as clinical risk signals, especially when they coincide with sustained warm nights, poor housing, limited cooling access, or high occupational exposure [19,20,75,76,77,78,96,97].

Second, exposure recognition should lead to biological and clinical risk assessment. In psychiatric populations, this assessment should consider thermoregulatory strain, hydration status, sleep disruption, renal vulnerability, medication burden, cognitive impairment, substance use, comorbid cardiovascular or renal disease, functional impairment, and capacity for behavioral thermoregulation [11,19,20,21,22,23,56,57,58,59,60,61,75,76,88,89,94]. This stage is essential because the same heat exposure may have different clinical implications depending on diagnosis, age, medication profile, housing, social support, and access to cooling. For example, a patient receiving lithium may require attention to hydration, renal function, and toxicity symptoms [22,23,60,61,89], whereas a patient with severe mental illness living alone in poorly cooled housing may require caregiver or community outreach rather than medication-focused monitoring alone [21,22,23,88,94,97].

Third, risk assessment should guide targeted psychiatric actions. These actions may include anticipatory heat counseling, medication safety review, hydration planning, sleep-protection advice, monitoring for delirium or toxicity symptoms, caregiver coordination, and proactive follow-up of high-risk patients [22,23,56,57,58,59,60,61,85,89,94,95]. These interventions should be proportionate to risk. For many patients, low-intensity preventive advice may be sufficient, whereas more structured monitoring may be appropriate for individuals with severe mental illness, bipolar disorder treated with lithium, neurocognitive disorders, substance use disorders, advanced age, high anticholinergic or sedative burden, or limited access to cooling [21,22,23,56,57,58,59,60,61,85,88,89,94,95]. Importantly, the pathway does not imply routine medication discontinuation during hot weather. Rather, it supports individualized risk reduction, continuity of psychiatric treatment, and early recognition of heat-related clinical deterioration.

Fourth, psychiatric actions should be embedded within public health adaptation. The heat-health literature shows that warning systems and heat action plans are most useful when they combine timely alerts with clear communication, surveillance, outreach to vulnerable groups, cooling access, occupational protections, and evaluation of effectiveness [80,81,82,85,86,91,92,93,94,95,96,97,98,99]. For mental health services, this means that psychiatric prevention should be connected to heat-health warning systems, emergency preparedness plans, primary care, community mental health teams, pharmacies, caregivers, social services, and local cooling infrastructure [90,94,95,98,99]. Structural adaptation is especially important because heat vulnerability is shaped by housing, poverty, urban design, occupational conditions, social isolation, and healthcare capacity rather than by individual behavior alone [73,74,75,76,77,78,87,88,95,96,97].

The proposed translational pathway should therefore be interpreted as an evidence-informed implementation framework rather than as a fully tested psychiatric intervention model. Its purpose is to organize the practical translation of the biological model into clinical and public health action while acknowledging that direct psychiatric heat-intervention trials remain limited. In implementation research, outcomes such as acceptability, feasibility, adoption, fidelity, implementation cost, penetration, and sustainability are important for understanding whether interventions can be translated into routine practice [100]. This is especially relevant because climate change has broad implications for mental health and requires psychiatric responses that can be integrated into health services and public health systems [101]. Consistent with implementation-science frameworks, future studies should evaluate not only clinical outcomes, such as psychiatric symptoms, emergency visits, relapse prevention, medication safety, sleep, hydration, and delirium vulnerability, but also implementation outcomes, including reach, effectiveness, adoption, implementation, maintenance, contextual determinants, equity, and sustainability [100,102,103]. This would allow heat-related psychiatric prevention to move from conceptual modeling toward testable, scalable, and context-sensitive interventions.

## 19. Conclusions

This review addressed the fragmented multidisciplinary gap identified in the Introduction by organizing the evidence across five complementary levels. First, it synthesized biological mechanisms linking acute and chronic heat exposure with climate-related psychiatric outcomes. Second, it integrated evidence from climate medicine, neuroscience, psychiatry, sleep research, psychopharmacology, and public health into a unified biological framework. Third, it distinguished pathways supported by stronger human evidence from those supported mainly by experimental, preclinical, severe-spectrum, or indirect mechanistic evidence. Fourth, it connected these mechanisms with major psychiatric outcomes and susceptible population groups, while recognizing the heterogeneity of exposure indicators and lag structures across the epidemiological literature. Fifth, it translated this synthesis into implications for future research, clinical practice, prescribing safety, heat-health preparedness, and climate adaptation in mental health care.

Heat exposure is an increasingly important biological and clinical determinant of mental health in a warming environment. The evidence reviewed in this article suggests that heat should not only be understood as environmental exposure or a public health hazard but also as a multi-system biological stressor with psychiatric relevance. Through thermoregulatory strain, autonomic and cardiovascular activation, dehydration, neuroinflammation, oxidative stress, mitochondrial dysfunction, blood–brain barrier disruption, HPA-axis activation, sleep and circadian disruption, neurotransmitter alterations, and psychopharmacological vulnerability, heat may influence psychiatric stability across diagnostic categories [11,12,13,14,15,16,17,18,19,20,21,22,23,31,32,33,34,35,36,37,38,39,40,41,42,43,44,45,46,47,48,49,50,51,52,53,54,55,56,57,58,59,60,61].

At the same time, the biological response to heat should not be framed as uniformly harmful. Mild, short-term, and recoverable heat exposure may activate adaptive cellular and physiological responses, including heat shock protein expression, cellular repair, thermoregulatory adjustment, and acclimatization-related mechanisms [11,32,36]. These responses may help preserve cellular integrity and improve tolerance to transient thermal stress. However, this potentially protective dimension should be interpreted cautiously in psychiatric contexts, because direct human evidence linking mild heat adaptation to improved mental health outcomes remains limited. The key distinction is therefore between adaptive, time-limited heat exposure with adequate recovery and maladaptive heat stress that is severe, prolonged, repeated without nighttime recovery, or combined with dehydration, sleep disruption, medication vulnerability, comorbidity, or social disadvantage.

The relationship between heat and mental health is transdiagnostic but not uniform across different populations. Depression, anxiety, bipolar disorder, psychotic vulnerability, substance-related crises, cognitive dysfunction, delirium vulnerability, and suicide-related outcomes may each be influenced by different combinations of shared biological pathways, medication exposure, sleep disruption, behavioral adaptation, and contextual vulnerability [5,6,7,8,62,63,64,65,66,67,68,69,70,71,72]. This supports a model in which heat-related psychiatric outcomes emerge from interactions between environmental exposure, biological susceptibility, clinical status and social conditions.

Therefore, biologically grounded climate psychiatry must integrate mechanisms with context. Age, comorbidity, severe mental illness, medication burden, housing quality, occupational exposure, access to cooling, healthcare capacity, and social support can determine whether heat-related biological stress is buffered or amplified [73,74,75,76,77,78]. These modifiers are not peripheral to biology; they shape thermoregulation, hydration, sleep recovery, medication safety, and access to protective behaviors.

For clinicians, the core implication is that heat exposure should become part of routine psychiatric risk assessment and treatment planning. During heatwaves or periods of sustained high temperature, clinicians should consider heat-sensitive symptom worsening, sleep disruption, hydration status, medication burden, renal vulnerability, cognitive impairment, substance use, housing conditions, and access to cooling. Particular attention is warranted for patients with severe mental illness, older adults, individuals receiving lithium, antipsychotics, anticholinergic medications, sedatives, or complex polypharmacy, and those with limited capacity for self-care or early help-seeking. Practical clinical actions include anticipatory counseling, medication safety review, hydration and sleep guidance, monitoring for delirium or toxicity symptoms, and targeted follow-up for patients at high risk [22,23,56,57,58,59,60,61,73,74,75,76,77,78].

For public health decision-makers, the core implication is that heat-health preparedness should explicitly include mental health. Heat-health warning systems, emergency preparedness plans, primary care networks, psychiatric services, community outreach, and climate adaptation policies should identify people with mental disorders as a priority population. Public health responses should also address the social and environmental conditions that amplify biological heat vulnerability, including poor housing, urban heat islands, occupational exposure, social isolation, limited cooling access, and under-resourced health systems. Integrating mental health into heat-health action plans can support earlier risk identification, targeted communication, continuity of psychiatric care, safer medication management, and protection of socially vulnerable groups during extreme heat events [73,74,75,76,77,78,80,81,82].

Future research should move beyond epidemiological associations to mechanistic, longitudinal, and clinically actionable evidence. Priorities include improved exposure assessment, indoor and nighttime temperature measurements, biomarker integration, sleep and circadian monitoring, medication-specific studies, severe mental illness cohorts, risk prediction, implementation studies, and intervention development [80,81,82].

Overall, the psychiatric impact of maladaptive heat exposure represents a biological, clinical, and public health challenge. Understanding how heat affects the brain, body, and psychiatric outcomes can support earlier recognition, safer prescribing, better protection of vulnerable populations, and more effective adaptation strategies. As climate change intensifies heat exposure worldwide, psychiatry and mental health services must become part of the broader climate-health response.

## Figures and Tables

**Table 1:** Biological pathways linking heat exposure to mental health outcomes ^1^.

Biological Pathway	Heat-Related Biological Process	Potential Psychiatric Relevance	Examples of Relevant Outcomes	Key Research Gaps	Primary Evidence Base and Narrative Credibility
Thermoregulatory strain	Heat exposure challenges central thermoregulation, particularly the hypothalamic and autonomic pathways involved in heat dissipation, sweating, vasodilation, and behavioral adaptation [11,12,13,26,27].	It may increase fatigue, discomfort, irritability, reduce cognitive efficiency, and increase vulnerability to symptom exacerbation in individuals with limited physiological or behavioral adaptive capacity.	Anxiety symptoms, agitation, mood instability, impaired self-care, and heat-related emergency presentations.	There is a need for studies integrating ambient temperature, indoor heat, thermoregulatory markers, psychiatric symptoms, and behavioral adaptation.	Stronger human physiological and mechanistic evidence.
Autonomic and cardiovascular activation	Heat exposure increases cardiovascular workload, heart rate, skin blood flow, sweating, and fluid demand [11,28,29,30].	Bodily sensations related to heat stress may overlap with or amplify anxiety, panic-like symptoms, somatic distress, and stress-related arousal.	Anxiety, panic vulnerability, somatic symptom burden, fatigue, and acute distress.	Limited psychiatric studies have measured autonomic responses during real-world heat exposure.	Stronger human physiological and experimental evidence.
Neuroinflammation	Heat stress and severe hyperthermia may activate systemic inflammatory pathways and neuroimmune signaling involving cytokines, microglia, astrocytes, and endothelial cells [14,15,31,32,33,34,35].	Inflammatory signaling may influence mood, motivation, cognition, fatigue, reward processing, and stress sensitivity.	Depressive symptoms, fatigue, cognitive dysfunction, irritability, and stress-related symptom worsening.	Few human studies have linked heat exposure, inflammatory biomarkers, and psychiatric outcomes in the same design.	Mixed evidence; mainly severe heat illness, experimental, cellular, and indirect psychoneuroimmunology evidence.
Oxidative stress	Heat stress can increase reactive oxygen species and oxidative imbalance, potentially damaging proteins, lipids, DNA, mitochondria, and cellular membranes [16,36,37,38].	Redox imbalance may contribute to fatigue, cognitive dysfunction, neuroplasticity impairment, and mood disorders.	Depression, bipolar disorder vulnerability, cognitive symptoms, and treatment-resistant phenotypes.	Longitudinal studies are needed to evaluate oxidative stress markers during heatwaves or repeated heat exposure.	Mainly experimental and indirect mechanistic evidence, supported by psychiatric oxidative-stress literature.
Mitochondrial dysfunction	Heat stress may disrupt the mitochondrial membrane potential, oxidative phosphorylation, redox balance, and immune signaling [36,39].	Impaired cellular energy regulation may contribute to fatigue, reduced resilience, cognitive inefficiency, and affective instability.	Fatigue, depressive symptoms, mood instability, and reduced stress tolerance.	There is a lack of psychiatric studies assessing mitochondrial markers in relation to heat exposure.	Mainly experimental, cellular, and indirect mechanistic evidence.
Blood–brain barrier and neurovascular dysfunction	Heat exposure and hyperthermia may compromise blood–brain barrier integrity through endothelial injury, inflammation, oxidative stress, and altered cerebral perfusion [17,18,31,40,41].	Increased barrier permeability may amplify central inflammatory signaling and disrupt brain homeostasis.	Cognitive dysfunction, delirium vulnerability, affective instability, and neuropsychiatric symptoms during severe heat stress.	There is a need for translational studies using neurovascular biomarkers, neuroimaging, and psychiatric assessments.	Mainly heatstroke, animal, in vitro, and neurovascular mechanistic evidence.
HPA-axis and stress biology	Heat exposure can act as a physiological stressor, engaging neuroendocrine and allostatic systems [42,43,44,45].	Repeated heat exposure may contribute to allostatic load, stress sensitivity, emotional dysregulation, and reduced recovery.	Anxiety, depressive symptoms, irritability, stress-related relapse, and sleep disturbance.	There is limited evidence on cortisol, allostatic load biomarkers, and psychiatric outcomes during chronic or recurrent exposure to heat.	Mixed human stress-biology and indirect mechanistic evidence.
Sleep and circadian disruption	Elevated nighttime temperatures may impair heat dissipation, delay sleep onset, reduce sleep duration, and disturb sleep architecture and circadian stability [19,20,46,47,48,49,50,51].	Sleep loss can reduce emotional regulation, increase irritability, worsen mood symptoms, and increase relapse vulnerability.	Depression, anxiety, bipolar relapse, psychotic vulnerability, and impaired daytime functioning.	Future research should incorporate nighttime temperature, indoor heat, actigraphy, sleep timing, and mental health outcomes.	Stronger human evidence from wearable, observational, experimental, and sleep-medicine studies.
Neurotransmitter alterations	Heat stress may influence the serotonergic, dopaminergic, noradrenergic, GABAergic, and glutamatergic systems involved in arousal, fatigue, motivation, mood, cognition, and thermoregulation [52,53,54,55].	Neurochemical shifts may contribute to fatigue, irritability, reduced cognitive control, anxiety-like arousal, and behavioral dysregulation.	Mood symptoms, anxiety, agitation, cognitive impairment, and reduced behavioral adaptation.	Direct evidence from psychiatric cohorts exposed to heat remains limited.	Limited and indirect evidence, mainly from heat-stress, exercise physiology, and experimental studies.
Psychopharmacological vulnerability	Psychotropic medications may modify heat vulnerability through their effects on sweating, thirst, sedation, cognition, autonomic regulation, renal function, and thermoregulation [22,23,56,57,58,59,60,61].	Medication-related vulnerability may increase the risk of dehydration, heat intolerance, delirium, lithium toxicity, and reduced behavioral adaptation during heatwaves.	Severe mental illness exacerbation, heat-related hospitalization, medication toxicity, and relapse risk.	There is a need for medication-specific studies under realistic environmental heat conditions, especially in older adults and low-resource settings.	Moderate human clinical, pharmacoepidemiological, and pharmacological evidence.

^1^ HPA, hypothalamic–pituitary–adrenal. The pathways summarized in this table are not mutually exclusive and may interact through inflammatory, oxidative, neuroendocrine, sleep-related, and pharmacological mechanisms. Evidence credibility was classified narratively as higher, moderate, or lower according to the primary evidence base available for each pathway, the extent of direct human evidence, and the degree of reliance on experimental, preclinical, or indirect mechanistic evidence. This classification was not intended to replace formal GRADE or risk-of-bias assessment.

**Table 2:** Heat-related vulnerability in major psychiatric disorders ^1^.

Psychiatric Condition or Outcome	Heat-Sensitive Mechanisms	Medication-Related Considerations	Clinical and Public health Implications	Selected Supporting Evidence
Depression and psychological distress	Heat-related sleep disruption, fatigue, inflammatory signaling, oxidative stress, reduced activity, discomfort, and reduced coping capacity may contribute to the development of depressive symptoms and psychological distress.	Antidepressants may affect sweating, autonomic tone, sleep, and hydration, depending on the class and individual vulnerability. The anticholinergic burden may impair heat dissipation.	During high-temperature periods, clinicians should assess sleep, fatigue, hydration, medication tolerability, social isolation, and functional deterioration of patients.	Systematic reviews and population studies have reported associations between elevated temperatures and poor mental health outcomes, including distress and mood-related presentations [5,6,7,8,62,63,70].
Anxiety, somatic distress, and stress-related symptoms	Heat-induced autonomic activation, sweating, palpitations, dizziness, bodily discomfort, sleep loss, and perceived threats may amplify anxiety and somatic symptoms.	Medications with sedative, anticholinergic, or autonomic effects may modify heat tolerance and behavioral responses.	Heatwaves may increase acute anxiety-related presentations, particularly among individuals with panic vulnerability, somatic symptom burden, trauma-related arousal, or limited access to cooling.	Emergency department studies report increased heat-related visits for anxiety, stress-related, and somatoform disorders; evidence from a South American emergency psychiatric setting also supports the relevance of temperature in psychiatric emergency consultations [62,64,65,72].
Bipolar disorder and mood instability	Disruption of sleep and circadian rhythm, nighttime heat, autonomic arousal, dehydration, and reduced recovery may destabilize mood regulation.	Lithium requires attention because dehydration, sodium balance, renal function, and fluid loss may affect the serum levels and toxicity risk. Antipsychotics, sedatives, and antidepressants may add to thermoregulatory or behavioral vulnerability.	Heat-risk counseling should include sleep protection, hydration, medication safety, recognition of early relapse signs, and monitoring during heatwaves.	Studies have linked ambient temperature with bipolar disorder admissions and youth mental health encounters; a South American case-crossover study found heat-related signals for bipolar disorder consultations at specific lag periods [64,66,72].
Schizophrenia-spectrum disorders and severe mental illness	Thermoregulatory impairment, cognitive dysfunction, reduced self-care, social isolation, sleep disruption, dehydration, and impaired behavioral thermoregulation may increase vulnerability.	Antipsychotics may impair thermoregulation through dopaminergic, anticholinergic, sedative, cardiovascular, and behavioral pathways of action. Polypharmacy may further increase this risk.	Heat-health plans should identify individuals with severe mental illness as a priority group for outreach, hydration support, medication review, and community monitoring.	Systematic evidence indicates increased vulnerability among individuals with mental illness during extreme heat; population studies report heat-related emergency visits for schizophrenia-spectrum disorders, although disorder-specific direction and magnitude may vary by setting [21,22,23,62,64,72].
Substance use disorders	Heat may worsen sleep disruption, irritability, impulsivity, dehydration, exposure risk, and impaired judgment among individuals with substance use disorders. Substance use may also reduce behavioral adaptation and increase physiological risks.	Alcohol, sedatives, stimulants, opioids, and polysubstance use may interact with hydration, thermoregulation, cognition, and behavioral risks during high temperatures.	Heatwave planning should include outreach for individuals with substance use disorders, especially those experiencing homelessness, outdoor exposure, comorbid mental illnesses, or limited access to healthcare.	Extreme heat has been associated with substance use disorder-related emergency visits, and recent meta-analytic evidence supports increased emergency healthcare utilization during extremely high temperatures [62,67].
Sleep–wake and circadian disorders	Elevated nighttime temperatures may delay sleep onset, reduce sleep duration, impair sleep quality, and disrupt circadian stability.	Sedatives and hypnotics can reduce alertness and behavioral responsiveness during heat exposure. Other psychotropics may indirectly affect sleep and thermal comfort.	Sleep-focused adaptation strategies, including access to cooling, nighttime heat monitoring, and insomnia management, may reduce psychiatric vulnerability.	Wearable-based and systematic review evidence shows that warmer nights erode sleep globally and that heat can impair sleep quantity and quality [19,20,46,47,48,49].
Neurocognitive and delirium vulnerability	Heat-related dehydration, electrolyte imbalance, cardiovascular strain, neuroinflammation, oxidative stress, and blood–brain barrier dysfunction may affect cognition and increase the risk of acute confusion in medically vulnerable individuals.	Anticholinergic burden, sedatives, lithium, polypharmacy, and medications affecting blood pressure or renal function may increase vulnerability.	Older adults and medically complex psychiatric patients require proactive monitoring during heatwaves, especially when they live alone or in poorly cooled environments.	Mechanistic evidence links heat stress to neurovascular dysfunction, inflammation, oxidative stress, and neurological consequences of hyperthermia [17,18,31,36,40,41].
Suicide mortality and self-harm-related outcomes	Heat may contribute to population-level risks through sleep disruption, distress, irritability, impulsivity, substance-related crises, autonomic arousal, and psychosocial stress.	Medication-related sedation, toxicity, withdrawal, and poor adherence may indirectly increase vulnerability during heat-related crises.	Heat-health warning systems should integrate mental health surveillance, crisis care access, outreach to high-risk groups, and sleep protection strategies.	Systematic reviews and meta-analyses have reported associations between high ambient temperatures and suicide mortality or self-harm-related healthcare encounters [6,7,9,62,68,69].

^1^ The categories are not mutually exclusive. Heat-related vulnerability may be transdiagnostic and reflect interactions among biological mechanisms, medication exposure, behavioral thermoregulation, comorbid medical illnesses, and social determinants. “Self-harm-related outcomes” is used here as an epidemiological and public health category.

**Table 3:** Future research agenda for heat, the brain, and mental health ^1^.

Research Priority	Recommended Approach	Key Biological or Clinical Variables	Expected Contribution	Selected Supporting References
Mechanistic longitudinal studies	Prospective and repeated-measures psychiatric cohort studies before, during, and after heatwaves or high-temperature periods, including recovery periods.	Inflammatory markers, oxidative stress markers, cortisol, sleep, hydration, renal function, autonomic measures, medication exposure, contextual vulnerability, and psychiatric symptoms.	Clarify whether heat exposure produces measurable biological changes linked to psychiatric outcomes and determine temporal relationships between exposure, biological response, and psychiatric deterioration.	[14,15,16,17,18,21,22,23,31,32,33,34,35,36,37,38,39,40,41,42,43,44,45,56,57,58,59,60,61,75,76,77,78,79]
Improved exposure assessment	Combine meteorological data with indoor temperature, nighttime heat, humidity, geolocation, wearables, and housing information.	Indoor heat, nighttime minimum temperature, apparent temperature, humidity, activity, and cooling access.	Identify biologically relevant heat exposure rather than relying only on outdoor ambient temperatures.	[11,19,20,75,76,77,78,79]
Wearable and indoor heat monitoring	Use wearable devices, actigraphy, heart-rate monitoring, personal thermal exposure measures, and indoor temperature sensors.	Sleep duration, sleep timing, activity, heart rate, physiological recovery, nighttime indoor temperature, household heat, and personal thermal exposure.	Capture biologically relevant exposure and recovery patterns more accurately than outdoor ambient temperature alone.	[19,20,46,47,48,49,50,51,75,76,77,78,79]
Sleep and circadian pathways	Use actigraphy, sleep diaries, circadian timing measures, and symptom tracking during hot periods.	Sleep duration, sleep onset, sleep efficiency, circadian regularity, mood, anxiety, irritability, and relapse indicators.	Test whether sleep disruption mediates heat-related psychiatric vulnerability.	[19,20,46,47,48,49,50,51]
Psychopharmacology and medication safety	Medication-specific observational studies and heat exposure studies in clinical populations.	Anticholinergic burden, lithium levels, renal function, hydration, sweating, sedation, orthostasis, and polypharmacy.	Identify medication-related heat risks and develop practical monitoring recommendations.	[22,23,56,57,58,59,60,61]
Severe mental illness	Clinical cohort studies of schizophrenia-spectrum disorders, bipolar disorder, and severe mood disorders during heat events.	Diagnosis, symptom severity, cognition, self-care, medication profile, comorbidity, housing, and emergency visits.	Determine the mechanisms of heat vulnerability in high-risk psychiatric populations.	[21,22,23,62,64,66,72]
Biomarker integration	Multi-marker studies combining immune, oxidative, endocrine, autonomic, renal, and sleep markers, using multimodal biomarker panels when feasible.	IL-6, CRP, TNF-α, cortisol, heart rate variability, oxidative stress markers, renal function, sleep metrics, hydration markers, neurovascular indicators, and medication-specific measures such as lithium concentrations when clinically appropriate.	Develop biological signatures of heat-related psychiatric vulnerability and identify candidate pathways for risk stratification and intervention studies.	[14,15,16,17,18,31,32,33,34,35,36,37,38,39,40,41,42,43,44,45,60,61]
Neuroimaging and neurovascular assessment	Use structural, functional, or neurovascular imaging in selected high-risk psychiatric cohorts exposed to recurrent heat.	Neurovascular markers, blood–brain barrier-related indicators, cerebral perfusion, inflammatory or structural brain changes, cognition, and psychiatric symptoms.	Test whether recurrent heat exposure is associated with measurable brain or neurovascular changes in vulnerable populations.	[18,40,41]
Prediction and risk stratification	Develop and externally validate risk models for psychiatric deterioration during heatwaves.	Age, diagnosis, medication exposure, comorbidities, prior admissions, housing, social support, and heat exposure.	Support targeted outreach and clinical prioritization during heat-health alerts.	[62,63,64,65,72,79,84]
Low- and middle-income settings	Conduct context-specific cohort studies, surveillance systems, and feasible intervention trials.	Heat exposure, housing, cooling access, occupational heat, service availability, medication continuity, and outcomes.	Generate evidence from regions with high heat vulnerability and limited research representation.	[2,3,72,73,74,75,76,77,78]
Intervention development	Develop and evaluate psychiatric heat-risk protocols using complex intervention frameworks, including intervention studies of feasible heat mitigation strategies in psychiatric populations.	Medication review, hydration planning, sleep protection, caregiver outreach, community cooling, service alerts, access to cooling, heat-risk counseling, and service-level heatwave protocols.	Translate biological understanding into prevention and clinical practice by evaluating effects on psychiatric symptoms, emergency visits, relapse prevention, medication safety, physiological outcomes, and acceptability.	[22,23,56,57,58,59,60,61,80,81,82,83]
Policy integration	Embed mental health indicators into heat-health warning systems and climate-resilient health systems.	Psychiatric emergency visits, medication complications, severe mental illness outreach, and crisis service demand.	Align psychiatric care with climate adaptation and public health preparedness.	[3,21,22,23,80,81,82]

^1^ CRP, C-reactive protein; IL-6, interleukin-6; TNF-α, tumor necrosis factor-alpha. The proposed agenda emphasizes integrated exposure, biological, clinical, and contextual measurements.

## Data Availability

No new data were created or analyzed in this study. Data sharing is not applicable to this article.

## Supplementary Materials

The following supporting information can be downloaded at: https://www.mdpi.com/article/10.3390/biology15141165/s1, Supplementary Table S1. Three-dimensional stratification of psychotropic medication-related heat vulnerability by medication intensity, regimen complexity, and patient vulnerability; Supplementary Table S2. Outcome-stratified summary of heat-exposure indicators and lag or effect windows in key epidemiological studies of heat-related mental health outcomes.

## Abbreviations

The following abbreviations are used in this manuscript:

BBB	Blood–brain barrier
CRP	C-reactive protein
DNA	Deoxyribonucleic acid
GABA	Gamma-aminobutyric acid
HPA	Hypothalamic–pituitary–adrenal
IL-6	Interleukin-6
ROS	Reactive oxygen species
TNF-α	Tumor necrosis factor-alpha
