# Supplementary material for: Heat, Brain, and Mental Health: Biological Mechanisms Underlying Climate-Related Psychiatric Outcomes
Source: Biology (Basel). 2026 Jul 16;15(14):1165. doi: 10.3390/biology15141165 (PMC13404331; doi:10.3390/biology15141165)
Supplement: Supplementary file 1 [file biology-15-01165-s001.zip › biology-4412554-supplementary.pdf]

Supplementary Material

Heat, Brain, and Mental Health: Biological Mechanisms Underlying Climate-Related Psychiatric Outcomes

Julio Torales [1,2,3,*], Iván Barrios [3,4], Marcelo O'Higgins [1], Tomás Caycho-Rodríguez [5], Antonio Ventriglio [6] and João Mauricio Castaldelli-Maia [7]

1 Grupo de Investigación sobre Epidemiología de los Trastornos Mentales, Psicopatología y Neurociencias, Facultad de Ciencias Médicas, Universidad Nacional de Asunción, San Lorenzo 111421, Paraguay; marcelo.g.ohiggins@gmail.com
2 Vicerrectoría de Investigación y Postgrado, Universidad de Los Lagos, Osorno 5290000, Chile
3 Facultad de Ciencias de la Salud, Universidad Sudamericana, Pedro Juan Caballero 130114, Paraguay; ivanjuan2013@gmail.com
4 Cátedra de Bioestadística, Filial Santa Rosa del Aguaray, Facultad de Ciencias Médicas, Universidad Nacional de Asunción, Santa Rosa del Aguaray 021801, Paraguay
5 Facultad de Psicología, Universidad Científica del Sur, Lima 15067, Peru; tcaycho@cientifica.edu.pe
6 Department of Clinical and Experimental Medicine, University of Foggia, Foggia 71121, Italy; a.ventriglio@libero.it
7 Department of Psychiatry, University of São Paulo, São Paulo 05403903, SP, Brazil; jmcmaia2@gmail.com
* Correspondence: juliotorales@gmail.com; Tel.: +595-971683748

Supplementary Table S1. Three-dimensional stratification of psychotropic medication-related heat vulnerability by medication intensity, regimen complexity, and patient vulnerability. [1]

Psychotropic category	Medication intensity	Regimen complexity	Patient vulnerability and comorbidities	Main heat-related risk boundaries	Clinical interpretation
Antipsychotics	Higher doses, recent dose escalation, high dopamine D2 blockade, anticholinergic or sedative properties, and orthostatic effects [22,23,56–59].	Antipsychotic polypharmacy; combination with anticholinergics, sedatives, antihypertensives, or other medications affecting autonomic function [22,23,56–59].	Severe mental illness, older age, cognitive impairment, cardiovascular disease, poor hydration, limited self-care, poor housing, and reduced cooling access [22,23,56–59].	Impaired thermoregulation, reduced behavioral adaptation, sedation, dehydration risk, delirium vulnerability, and rare hyperthermic syndromes [22,23,56–59].	Risk is highest when pharmacological burden, severe illness, impaired self-care, and environmental heat overlap [22,23,56–59].
Antidepressants	Higher doses, recent dose escalation, anticholinergic load, serotonergic or noradrenergic effects, and sedative properties [22,23,57–59].	Combination with other serotonergic agents, anticholinergics, sedatives, diuretics, or medications affecting blood pressure or hydration [22,23,57–59].	Older age, cardiovascular disease, autonomic vulnerability, dehydration risk, cognitive impairment, polypharmacy, and poor cooling access [22,23,57–59].	Altered sweating, autonomic symptoms, orthostatic vulnerability, sedation, cognitive impairment, and rare serotonin-toxicity-related hyperthermia [22,23,57–59].	Risk varies by agent and is greatest with anticholinergic burden, polypharmacy, dehydration, and frailty [22,23,57–59].
Lithium	High dose, recent dose increase, high-normal serum concentration, and narrow therapeutic range [22,23,60,61].	Combination with medications affecting renal function, sodium balance, hydration, or lithium clearance [22,23,60,61].	Renal impairment, older age, dehydration, vomiting, diarrhea, reduced oral intake, cardiovascular disease, and limited laboratory monitoring [22,23,60,61].	Lithium accumulation or toxicity, dehydration-related renal vulnerability, confusion, tremor, gastrointestinal symptoms, and delirium vulnerability [22,23,60,61].	Heat risk is not an inevitable seasonal increase in lithium levels; it is highest when heat exposure coincides with dehydration, renal vulnerability, interacting medications, or limited monitoring [22,23,60,61].
Other mood stabilizers	Higher doses, sedative or cognitive adverse effects, and hepatic or metabolic burden depending on the agent [22,23,56–61].	Combination with antipsychotics, sedatives, antidepressants, or other medications increasing cognitive or sedative burden [22,23,56–61].	Neurological vulnerability, hepatic disease, metabolic disease, cognitive impairment, substance use, and dehydration risk [22,23,56–61].	Sedation, dizziness, cognitive slowing, impaired heat avoidance behavior, and reduced self-care [22,23,56–61].	Risk is mainly indirect and depends on sedation, cognition, comorbidity, and combined medication burden [22,23,56–61].

Benzodiazepines and sedative-hypnotics	Higher doses, long half-life agents, recent initiation or escalation, and daytime sedation [22,23,57–59].	Combination with alcohol, opioids, antipsychotics, antidepressants, antihistamines, or other sedatives [22,23,57–59].	Older age, cognitive impairment, substance use, respiratory disease, poor housing, social isolation, and limited cooling access [22,23,57–59].	Sedation, impaired judgment, falls, reduced heat avoidance behavior, and dehydration risk [22,23,57–59].	Risk is highest when sedation reduces behavioral thermoregulation or the ability to seek help during heat exposure [22,23,57–59].
Substance-related or non-prescribed psychoactive exposures	Dose and pattern of use, including stimulant, sedative, alcohol, or polysubstance exposure [22,23,57–59].	Polysubstance use; combination with prescribed psychotropics; concurrent dehydration or sleep deprivation [22,23,57–59].	Substance use disorders, homelessness, poor nutrition, limited access to care, occupational heat exposure, and social vulnerability [22,23,57–59].	Dehydration, autonomic activation, impaired judgment, sleep loss, agitation, intoxication, withdrawal, and heat illness risk [22,23,57–59].	Risk is strongly context-dependent and may be amplified by unstable housing, poor hydration, intoxication, withdrawal, or limited access to care [22,23,57–59].

[1] The stratification presented in this table is based on the psychopharmacological and heat-related vulnerability evidence discussed in Section 10 and should be interpreted as a clinical synthesis rather than as a formal medication-specific risk score [22,23,56–61].

Supplementary Table S2. Outcome-stratified summary of heat-exposure indicators and lag or effect windows in key epidemiological studies of heat-related mental health outcomes. [1]

Mental health outcome or diagnostic category	Representative evidence	Population or setting	Heat-exposure indicator	Exposure contrast or definition	Lag or effect window	Standardized comparison notes
Transdiagnostic mental health-related emergency department visits	Nori-Sarma et al. [62]; Mullins and White [63]	US adults with commercial or Medicare Advantage insurance; California and broader US datasets	Daily maximum ambient temperature in Nori-Sarma et al.; ambient temperature measures in Mullins and White	Extreme heat defined as the 95th percentile of the county-specific temperature distribution versus the optimal temperature in Nori-Sarma et al.; hot versus colder temperature ranges in Mullins and White	Nori-Sarma et al.: distributed lag structure up to 5 days, with the strongest increase on lag 0 and some persistence at lags 2–4. Mullins and White: contemporaneous and cumulative exposure windows, including longer windows up to several months.	This outcome is best compared as acute service utilization. Exposure metrics differ across studies, so effect sizes should not be pooled directly without harmonization.
Depression, psychological distress, and poor mental health days	Mullins and White [63]; Thompson et al. [7]; Liu et al. [5]	Population-based US data and systematic review evidence across settings	Ambient temperature, including mean temperature or temperature-bin approaches depending on the study; heterogeneous metrics in systematic reviews	Higher temperatures compared with cooler/reference temperature ranges; one-degree changes in mean temperature in some analyses	Mullins and White evaluated contemporaneous and cumulative windows; systematic reviews included heterogeneous lag structures and exposure definitions.	Use this row descriptively. The exposure metric is not equivalent across studies; depression-related outcomes include self-reported distress, poor mental health days, depressive symptoms, and service use.
Anxiety, stress-related, somatoform, and emotional dysregulation outcomes	Nori-Sarma et al. [62]; Basu et al. [65]; Niu et al. [64]	US adults; California emergency room visits; children, adolescents, and young adults in New York City	Daily maximum temperature in Nori-Sarma et al.; same-day mean apparent temperature in Basu et al.; daily minimum temperature as a nighttime heat indicator in Niu et al.	Nori-Sarma et al.: 95th percentile daily maximum temperature versus optimal temperature. Basu et al.: 10 °F increase in same-day mean apparent temperature. Niu et al.: elevated minimum temperature, 95th percentile, versus minimum risk temperature.	Nori-Sarma et al.: lag up to 5 days, strongest at lag 0. Basu et al.: same-day exposure. Niu et al.: cumulative lag 0–5 days.	This category includes both diagnostic and symptom-related service-use outcomes. Apparent temperature incorporates humidity and should not be treated as directly equivalent to maximum or minimum temperature.
Bipolar disorder and mood instability	Sung et al. [66]; Niu et al. [64]; Torales et al. [72]	Taiwan national psychiatric inpatient cohort; New York City youth encounters; psychiatric emergency consultations in Paraguay	Daily ambient temperature in Sung et al.; daily minimum temperature in Niu et al.; daily mean temperature in Torales et al.	Sung et al.: increasing risk above approximately 24 °C and greatest risk at extreme high daily temperatures. Niu et al.: 95th percentile daily minimum temperature versus minimum risk temperature. Torales et al.: temperature percentiles and diagnosis-specific odds ratios.	Sung et al.: daily temperature association. Niu et al.: cumulative lag 0–5 days. Torales et al.: seven-day distributed lag approach with lag 1, lag 3, and lag 7 estimates reported.	Bipolar outcomes are particularly sensitive to sleep and circadian mechanisms, but comparisons are complicated by different outcome settings: inpatient admissions, youth encounters, and emergency psychiatric consultations.
Schizophrenia-spectrum disorders and psychosis-related outcomes	Nori-Sarma et al. [62]; Niu et al. [64]; Torales et al. [72]	US adult emergency department visits; New York City youth encounters; psychiatric emergency consultations in Paraguay	Daily maximum temperature in Nori-Sarma et al.; daily minimum temperature in Niu et al.; daily mean temperature in Torales et al.	Nori-Sarma et al.: 95th percentile daily maximum temperature versus optimal temperature. Niu et al.: 95th percentile daily minimum temperature versus minimum risk temperature. Torales et al.: percentile-based daily mean temperature exposure.	Nori-Sarma et al.: lag up to 5 days, with no clear diagnosis-specific lag pattern. Niu et al.: cumulative lag 0–5 days. Torales et al.: lag 1, lag 3, and lag 7 estimates.	Psychosis-related findings should be interpreted alongside medication exposure, behavioral thermoregulation, access to care, and possible changes in help-seeking during heat.

Substance use disorders and substance-related emergency healthcare utilization	Nori-Sarma et al. [62]; Jhang et al. [67]	US adult emergency department visits; systematic review and meta-analysis of emergency healthcare use for substance use disorders	Daily maximum temperature in Nori-Sarma et al.; mean ambient temperature or study-specific extreme temperature metrics in Jhang et al.	Nori-Sarma et al.: 95th percentile daily maximum temperature versus optimal temperature. Jhang et al.: extremely high ambient temperature commonly defined as the top 5% of the temperature distribution for ED visits.	Nori-Sarma et al.: lag up to 5 days, strongest overall at lag 0. Jhang et al.: lag structures varied across included studies.	Evidence is consistent with acute heat-related increases in substance-related emergency use, but heterogeneity by substance type, housing, comorbidity, and study design remains substantial.
Self-harm, suicide attempts, and suicide mortality	Nori-Sarma et al. [62]; Basu et al. [65]; Burke et al. [9]; Heo et al. [68]; Chen et al. [69]	US adult ED visits; California emergency room visits; US and Mexico suicide mortality; systematic review and meta-analysis evidence	Daily maximum temperature in Nori-Sarma et al.; same-day mean apparent temperature in Basu et al.; monthly average temperature in Burke et al.; short-term ambient temperature metrics in meta-analytic studies	Nori-Sarma et al.: 95th percentile daily maximum temperature versus optimal temperature. Basu et al.: 10 °F increase in same-day mean apparent temperature. Burke et al.: 1 °C increase in monthly average temperature.	Nori-Sarma et al.: lag up to 5 days. Basu et al.: same-day exposure. Burke et al.: monthly time scale. Meta-analyses included heterogeneous short-term lag structures.	This row combines acute self-harm service use and suicide mortality, which are not identical outcomes. Direct comparison requires attention to outcome severity and temporal scale.
Children, adolescents, and young adults with mental health-related ED or hospital encounters	Niu et al. [64]; Basu et al. [65]	New York City, ages 6–25 years; California emergency room visits including age-stratified analyses	Daily minimum temperature in Niu et al.; same-day mean apparent temperature in Basu et al.	Niu et al.: 95th percentile daily minimum temperature versus age-specific minimum risk temperature. Basu et al.: 10 °F increase in same-day mean apparent temperature.	Niu et al.: cumulative lag 0–5 days. Basu et al.: same-day exposure.	This category is included because developmental vulnerability was specifically highlighted in the review. Minimum temperature may capture nighttime heat and sleep-related mechanisms more directly than daytime maximum temperature.
Emergency psychiatric consultations in a South American low- and middle-income setting	Torales et al. [72]	Adults attending the psychiatric emergency service of a university hospital in Paraguay, 2021–2023	Daily mean temperature, with additional meteorological variables including humidity, pressure, precipitation, and sunshine duration	Temperature values represented by percentiles across the observed range; diagnosis-specific odds ratios estimated in relation to temperature exposure	Distributed lag non-linear model over a seven-day lag period; lag 1, lag 3, and lag 7 estimates reported for diagnostic groups.	This study adds LMIC and South American evidence but used daily mean temperature rather than maximum, minimum, or apparent temperature; therefore, comparison with other studies should remain descriptive.

[1] This supplementary table is intended to standardize comparison dimensions descriptively. Because the cited studies differ in exposure metric, temperature threshold, outcome definition, population, season, and lag structure, the table should not be interpreted as a quantitative ranking of effect sizes across diagnoses. ED, emergency department; MRT, minimum risk temperature.
